# PET Micro(nano)plastics Modulate Metformin–Albumin Binding and Species-Specific Bacterial Responses

**DOI:** 10.3390/ijms27167504

**Published:** 2026-08-21

**Authors:** Hasan Saygin, Elif Aydin, Asli Baysal

**Affiliations:** 1 Application and Research Center for Advanced Studies, Istanbul Aydin University, Sefakoy Kucukcekmece, Istanbul 34295, Türkiye; 2 Department of Molecular Biology and Genetics, Faculty of Science and Letters, Istanbul Technical University, Maslak, Sariyer, Istanbul 34469, Türkiye; aydineli22@itu.edu.tr; 3 Department of Chemistry, Faculty of Science and Letters, Istanbul Technical University, Maslak, Sariyer, Istanbul 34469, Türkiye

**Keywords:** metformin, polyethylene terephthalate, micro(nano)plastics, bovine serum albumin, protein binding, oxidative potential, reactive oxygen species, biofilm formation, *Escherichia coli*, *Staphylococcus aureus*

## Abstract

Metformin is a widely used antidiabetic drug that may enter biological and environmental systems together with micro/nanoplastics; however, their combined effects on protein interactions and microbial responses remain insufficiently understood. This study investigated how polyethylene terephthalate micro/nanoplastics (PET MNPs) influence metformin interactions with bovine serum albumin (BSA) and the subsequent responses of *Escherichia coli* and *Staphylococcus aureus*. BSA–metformin systems were conditioned with three PET MNP loads and increasing metformin concentrations. The resulting particle-depleted filtrates were evaluated using fluorescence spectroscopy, ultraviolet–visible spectroscopy, the Bradford assay, Rayleigh light scattering, turbidity, dithiothreitol-based oxidative potential, and reactive oxygen species (ROS) measurements. Bacterial growth, superoxide dismutase activity, glutathione-related thiol antioxidant response, lipid peroxidation, ROS generation, and biofilm formation were also assessed. PET MNP conditioning altered the fluorescence responses of tryptophan and tyrosine, modified BSA-associated absorbance, and produced non-linear changes in protein accessibility, aggregation-related scattering, turbidity, and oxidative indicators. The bacterial responses were species-specific. *Escherichia coli* showed increased bacterial growth under several exposure conditions, whereas *Staphylococcus aureus* exhibited reduced growth following metformin addition, particularly at the highest PET MNP load. *Staphylococcus aureus* also showed consistently elevated biofilm formation and a pronounced transient ROS increase under the high-PET, low-metformin condition. These findings indicate that upstream PET MNP conditioning can modify the physicochemical and biological properties of the filter-passing BSA–metformin phase, leading to concentration-dependent and species-specific bacterial responses.

## 1. Introduction

Microplastics (MPs) are generally defined as solid, water-insoluble plastic particles measuring ≤5 mm in size, whereas nanoplastics (NPs) refer to submicron plastic particles that are primarily generated through the fragmentation and degradation of larger plastic debris [1,2,3]. However, the definition of NPs remains under debate, with different studies using upper size limits of either 100 nm or 1000 nm, and no full consensus has yet been reached regarding their physicochemical characteristics [1,4]. Therefore, the term micro(nano)plastics (MNPs) represent an integrated terminology covering both microplastic and nanoplastic particles, and is increasingly used to describe plastic particles across the micro- and nanoscale continuum.

MNPs are widely distributed across aquatic, terrestrial, atmospheric, and biological environments because of diverse marine and land-based activities. Their presence has been reported not only in soil, water, and air, but also in marine organisms, salt, and bottled drinking water worldwide, indicating broad environmental and human exposure pathways [5,6,7]. Polyethylene terephthalate (PET)-derived MNPs are increasingly recognized as persistent contaminants in aquatic, terrestrial, atmospheric, and food-related environments [8]. Beyond environmental compartments, plastic particles, including PET, have been detected in various human biological matrices such as blood, placenta, feces, and breast milk, suggesting that human exposure occurs through multiple pathways [9]. However, the exact physiological burden remains difficult to quantify. As highlighted in recent comprehensive reviews, the detection of micro- and nanoplastics in complex human biological materials is currently constrained by significant methodological and instrumental limitations. These analytical challenges include the pervasive risk of background contamination, the lack of standardized extraction protocols, and the difficulty of definitively resolving nanoscale particles within protein- and lipid-rich samples [9]. Following ingestion or biological uptake, MNPs may also interact with biomolecules after entering biological systems. These findings highlight the need to understand how PET MNPs interact with biomolecules and modulate biological responses under realistic co-contamination scenarios. Considering the widespread occurrence of MNPs and their potential biological accessibility, their interaction with serum albumin in circulation represents an important mechanism for understanding possible health-related effects. Serum albumin is the most abundant protein in blood plasma and plays a central role in the transport of numerous endogenous and exogenous molecules. It is especially relevant because previous studies have shown that plastic particles can interact with albumin and induce conformational changes in the protein structure [10,11].

Human serum albumin (HSA) and bovine serum albumin (BSA) share high structural homology and are therefore widely used as model proteins in biochemical and biophysical interaction studies [12]. Albumin is a flexible multidomain carrier containing hydrophobic and polar binding regions that enable the reversible transport of drugs, fatty acids, hormones, and environmental contaminants [13,14]. Its aromatic amino acid residues also generate intrinsic fluorescence, allowing ligand binding and local conformational changes to be monitored spectroscopically [15,16,17]. In particular, the tryptophan (Trp) residue located in subdomain IIA is sensitive to changes in its surrounding microenvironment. Metformin binds weakly to moderately to albumin, mainly through hydrogen bonding and van der Waals interactions, and its proximity to the Trp-containing region can produce fluorescence quenching and modest conformational changes [18,19,20,21,22]. These combined binding and spectroscopic properties make albumin a relevant model for determining whether PET MNPs alter metformin–protein interactions.

Metformin is a first-line treatment for type 2 diabetes and may coexist with PET MNPs in biological compartments. Because serum albumin plays a central role in drug transport, while MNPs can modify protein structure and ligand accessibility, such co-exposure may alter metformin–albumin interactions and the composition of the surrounding exposure medium. In natural and biological environments, degrading commercial plastics inevitably leach inherent industrial additives into this medium, creating a complex mixture rather than a pure polymer suspension. *Escherichia coli* (*E. coli*) and *Staphylococcus aureus* (*S. aureus*) were therefore included as representative Gram-negative and Gram-positive bacterial models to determine whether these physicochemical changes within this complex aqueous phase are accompanied by species-specific effects on bacterial growth, oxidative stress, and biofilm formation [18,23,24,25].

Previous studies on polystyrene (PS) NPs provide a general mechanistic precedent for plastic–protein interactions. PS NPs have been reported to form ground-state complexes with serum albumin through hydrogen bonding, van der Waals forces, and hydrophobic interactions, resulting in changes in protein conformation and secondary structure [10,26]. Metformin adsorption onto PS NP surfaces has also been shown to modify subsequent interactions between the particles and albumin, possibly by partially occupying or shielding particle-surface binding regions [27]. These findings demonstrate that nanoplastic surfaces can act as interfacial platforms that influence both pharmaceutical adsorption and protein accessibility.

However, PS NPs cannot be considered direct predictive surrogates for PET MNPs because the interaction behavior of plastic particles depends on polymer-specific surface chemistry, particle size, charge, crystallinity, weathering state, and associated additives. PET contains chemically distinct aromatic ester groups and may therefore display different adsorption and protein-binding behavior from polystyrene. PET was selected in the present study because of its widespread occurrence in environmental, food-contact, and biological exposure pathways and because PET particles have previously been shown to interact with albumin and alter protein-associated structural and oxidative responses [11,28,29]. Furthermore, to accurately reflect authentic environmental exposure scenarios rather than idealized laboratory conditions, MNPs derived from commercial PET products were intentionally utilized. This approach accounts for the inherent industrial additives that leach into the aqueous phase during plastic fragmentation. Nevertheless, the specific influence of PET MNPs on metformin–albumin binding, and the possible downstream consequences for bacterial responses, has not been adequately investigated. Thus, the PS NP literature is used here as a mechanistic comparison, whereas the present study directly addresses the PET-specific knowledge gap. Despite growing evidence that micro- and nanoplastics of different polymer types, including PS, PVC, and PET, can interact with serum albumin [30,31,32,33], studies addressing pharmaceutical–plastic–protein interactions remain comparatively limited and have predominantly relied on PS-based model particles [33,34]. Consequently, the influence of environmentally relevant PET particles on drug–albumin interactions remains much less characterized. Since bacteria may encounter both pharmaceuticals and plastic particles in environmental and biological compartments, evaluating bacterial growth, oxidative stress, and biofilm formation provides an additional biological layer for understanding the relevance of these interactions.

Therefore, this study aimed to investigate the interactions within the three-component system of commercial PET MNPs, metformin, and BSA, and to specifically evaluate the biological endpoints modulated by the resulting aqueous phase in *E. coli* and *S. aureus*. Crucially, rather than isolating the direct physical effects of pristine particles, this study focuses on the authentic aqueous exposure medium, which encompasses both the physicochemical protein–drug interaction byproducts and the leachable additives inherent to commercial plastics. For this purpose, BSA–metformin systems were evaluated in the presence of different PET MNP loads using fluorescence spectroscopy, UV–vis analysis, Bradford assay, Rayleigh scattering, turbidity, DTT oxidative potential, and ROS measurements. In parallel, bacterial growth, oxidative stress markers, lipid peroxidation, and biofilm formation were assessed to compare Gram-negative and Gram-positive responses. This integrated approach links molecular-level protein interaction data with bacterial stress and adaptation outcomes under realistic, commercial PET MNP–pharmaceutical co-exposure conditions.

## 2. Results

### 2.1. Properties of Post-Filtration BSA–Metformin Systems Previously Conditioned with PET MNPs

**Figure 1:**
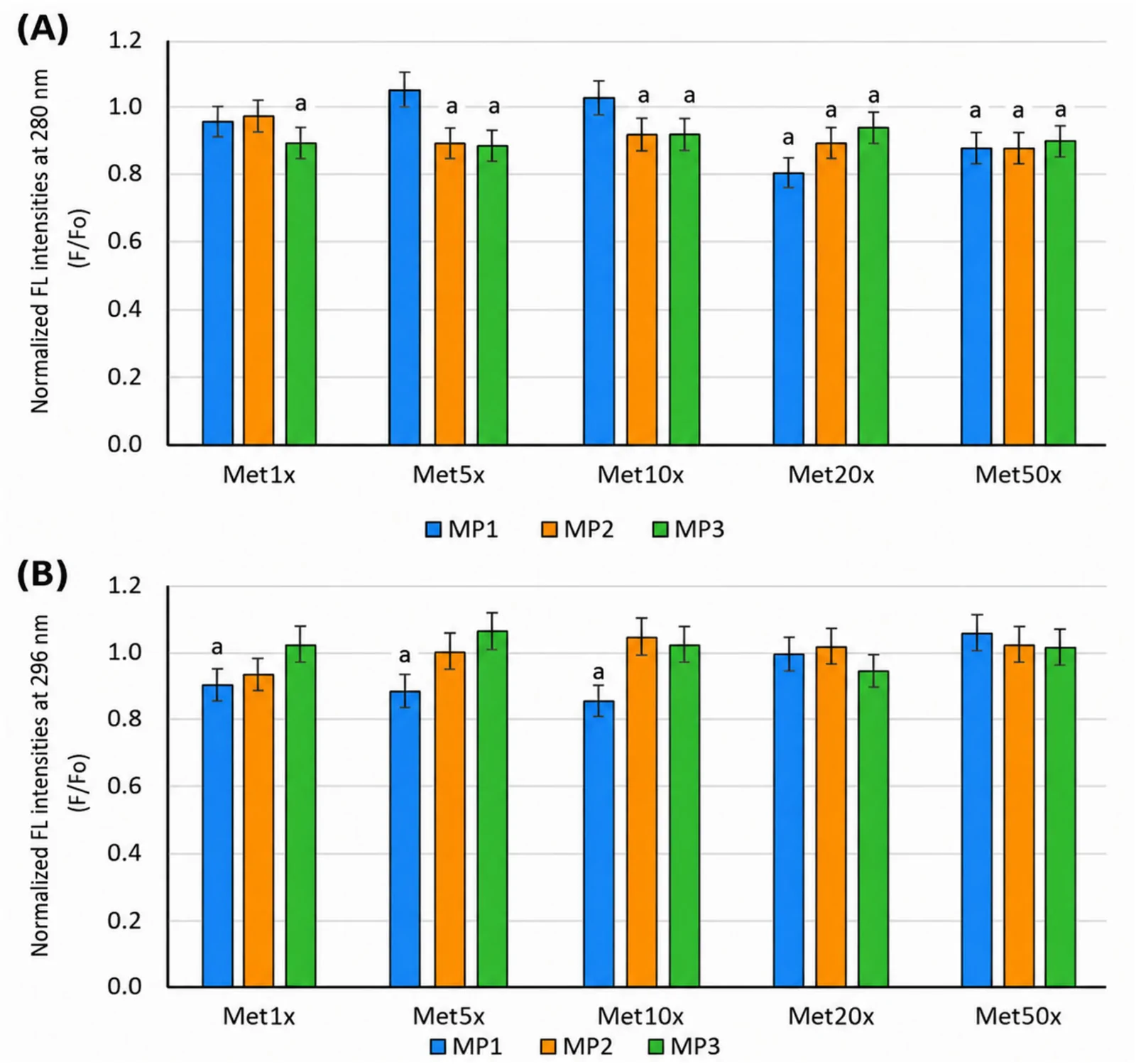
Normalized fluorescence intensities of BSA–metformin systems in the presence of different PET MNP concentrations. (**A**) Fluorescence intensity measured at an excitation wavelength of 280 nm and an emission wavelength of 340 nm. (**B**) Fluorescence intensity measured at an excitation wavelength of 296 nm and an emission wavelength of 311 nm. Fluorescence values were normalized to the corresponding controls (F/F_0_). MP1, MP2, and MP3 represent 0.5, 2.5, and 7.5 mg/mL PET MNPs, respectively. Data are presented as mean ± SD; error bars indicate the standard deviation. a: significantly different from control BSA and/or the corresponding group without MP (*p* < 0.05).

Trp-related normalized fluorescence showed PET-load- and metformin-concentration-dependent but non-monotonic changes (Figure 1A). In the MP1 set, fluorescence slightly increased at Met5x and Met10x, whereas lower values were observed at Met1x, Met20x, and Met50x. In the MP2 and MP3 sets, fluorescence values were generally below or close to the control level across metformin concentrations, indicating modest perturbation of the Trp microenvironment. However, the response was not strictly concentration-dependent, suggesting that PET MNP load and metformin concentration jointly influenced local aromatic residue accessibility. In conclusion, it can be said that the Trp micro-environment is affected by metformin and MP concentrations.

The results of the synchronous fluorescence spectroscopy analysis performed for Tyr residues are shown in Figure 1B. For MP1, it can be said that Met1x, Met5x, and Met10x concentrations decreased the fluorescence intensity, Met20x remained the same as the control, and there was an increase in Met50x. In the MP2 set, Met1x decreased fluorescence intensity while Met5x remained constant; Met10x, Met20x, and Met50x increased the intensity. In MP3 samples, while increases were observed in Met1x, Met5x, Met10x, and Met50x, the fluorescence intensity decreased in Met20x. Based on these results, it can be said that the presence of metformin and MP affects the Tyr microenvironment.

Comparative analysis using ANOVA confirmed that PET MNP load and metformin concentration significantly affected the spectroscopic responses of the BSA–metformin system (*p* < 0.05). In the Trp-related fluorescence measurements, significant reductions were observed especially in the MP2 and MP3 sets compared with the corresponding controls, indicating that higher PET MNP loads produced stronger perturbation of the Trp microenvironment. tyrosine (Tyr)-related fluorescence also differed significantly among exposure groups, but the response was less uniform, suggesting residue-specific and concentration-dependent conformational changes.

**Figure 2:**
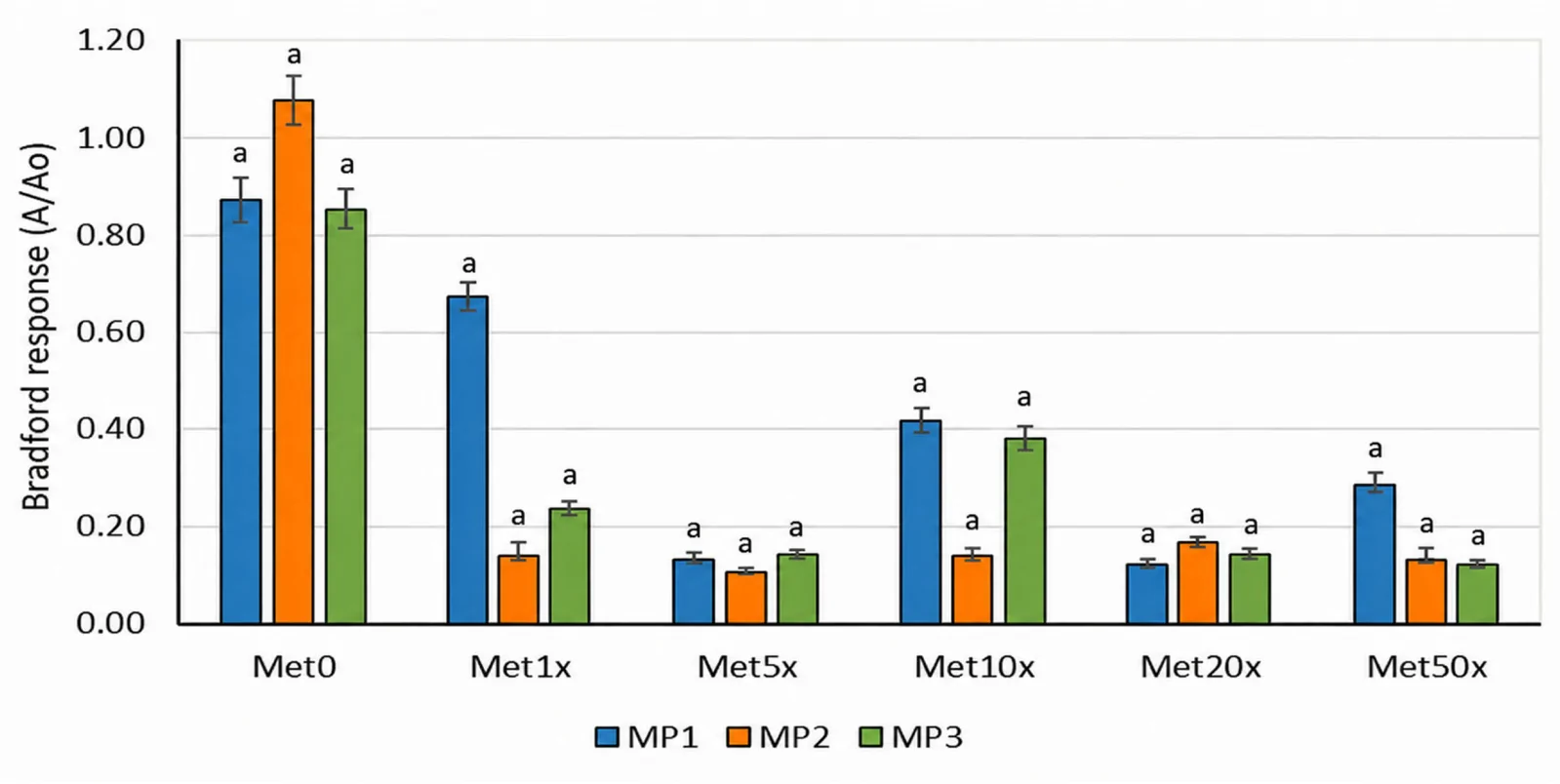
Bradford analyses results of BSA-metformin in the presence of PET MNPs, normalized to BSA. Data are presented as mean ± SD; error bars indicate the standard deviation. a: significantly different from control BSA and/or the corresponding group without MP (*p* < 0.05).

A significant decrease in the absorbance values at 595 nm by Bradford assay was observed in all samples upon the addition of metformin (*p* < 0.05); the results normalized to BSA are shown in Figure 2. Bradford protein assay results showed a clear decrease in normalized absorbance at 595 nm after PET MNP exposure in the BSA–MET system. In the Met0 control, all PET MNP groups showed the highest absorbance values, with MP2 slightly higher than MP1 and MP3. After Met1x exposure, MP1 retained a relatively higher signal, whereas MP2 and MP3 decreased markedly. At Met5x, all groups showed low absorbance values, with only minor differences among MP1, MP2, and MP3. At Met10x, MP1 and MP3 showed a partial increase compared with Met5x, while MP2 remained low. At Met20x and Met50x, absorbance values were generally low across all groups, although MP1 showed a slightly higher response at Met50x. Overall, the Bradford results indicate that PET MNP exposure reduced measurable BSA-associated protein signal in the presence of metformin, with the strongest decreases observed for MP2 and MP3 at most metformin doses (*p* < 0.05).

**Figure 3:**
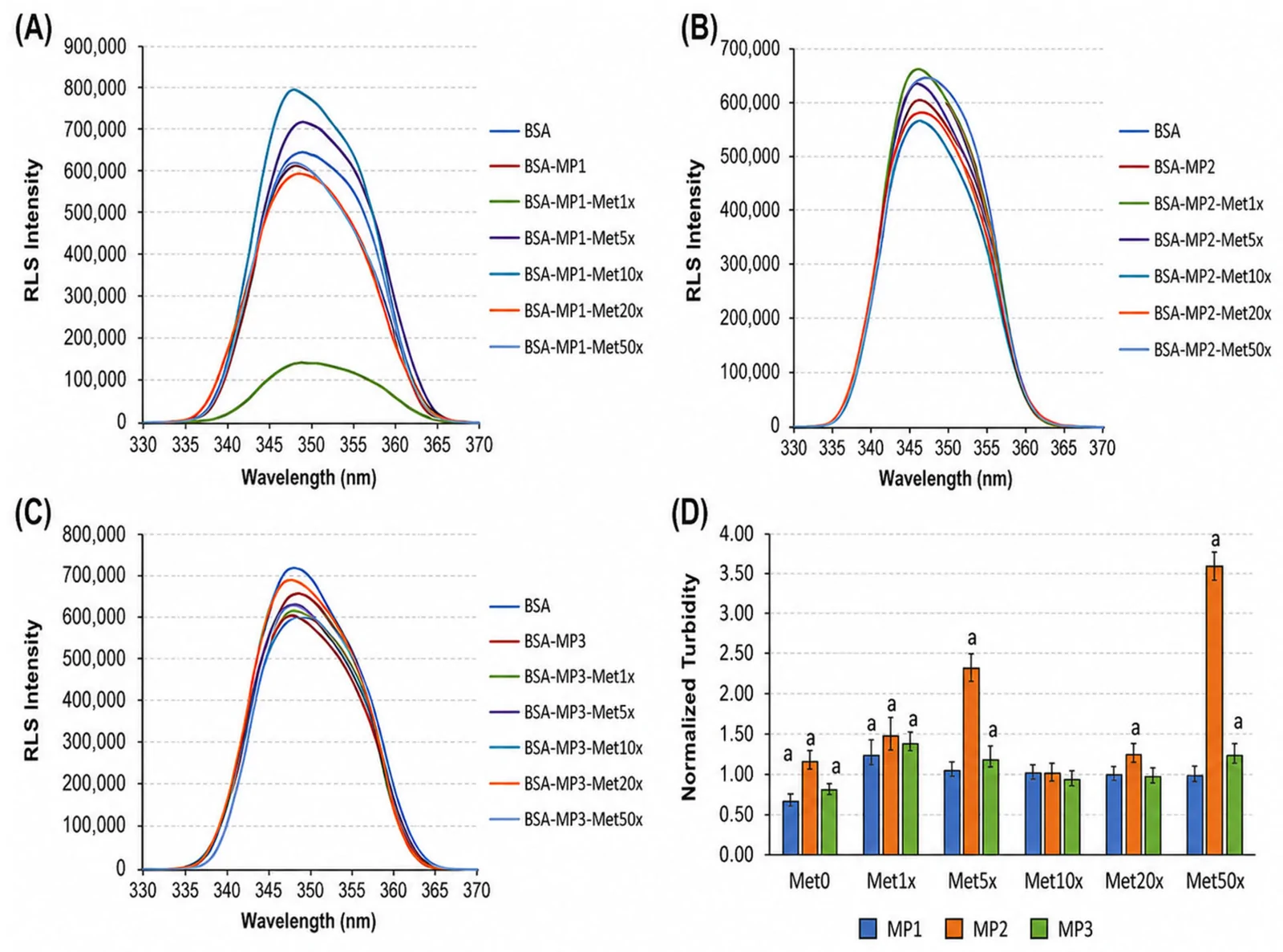
Rayleigh spectra for (**A**) MP1 set, (**B**) MP2 set, (**C**) MP3 set, and (**D**) turbidity ratio results. Data are presented as mean ± SD; error bars indicate the standard deviation. a: significantly different from control BSA and/or the corresponding group without MP (*p* < 0.05).

Rayleigh scattering spectra are presented in Figure 3A–C. Rayleigh spectra differed among the MP1, MP2, and MP3 sets. In the MP1 set, Met5x and Met10x increased the intensity, while Met1x, Met20x, and Met50x decreased the intensity (Figure 3A). As shown in Figure 3B, in the MP2 set; Met1x increased the intensity, while Met5x, Met10x, Met20x, and Met50x decreased it. Finally, in the MP3 set, Met1x and Met5x showed decreased intensity, while Met10x, Met20x, and Met50x showed increased intensity (Figure 3C).

Turbidimetric measurement results normalized to BSA are shown in Figure 3D. Normalized turbidity varied among the PET MNP groups in the metformin–BSA system. MP2 showed the highest turbidity response, increasing markedly at Met5x and reaching the maximum value at Met50x. MP1 remained close to baseline across all metformin concentrations, with only small changes. MP3 showed moderate increases, mainly at Met1x and Met50x. The overall turbidity response followed the order MP2 > MP3 > MP1 at the highest metformin level.

Comparative analysis also indicated that Rayleigh scattering and turbidity measurements showed significant differences among PET MNP loads and metformin concentrations (*p* < 0.05). The MP2 set exhibited significantly higher turbidity than MP1 at several metformin concentrations, particularly at Met5x and Met50x, supporting enhanced aggregation or light-scattering complex formation under medium PET MNP load. In contrast, MP1 generally remained close to baseline, indicating a weaker aggregation-related response.

### 2.2. UV–Vis Evidence of Aromatic Residue Microenvironment Changes in the BSA–Metformin System

**Figure 4:**
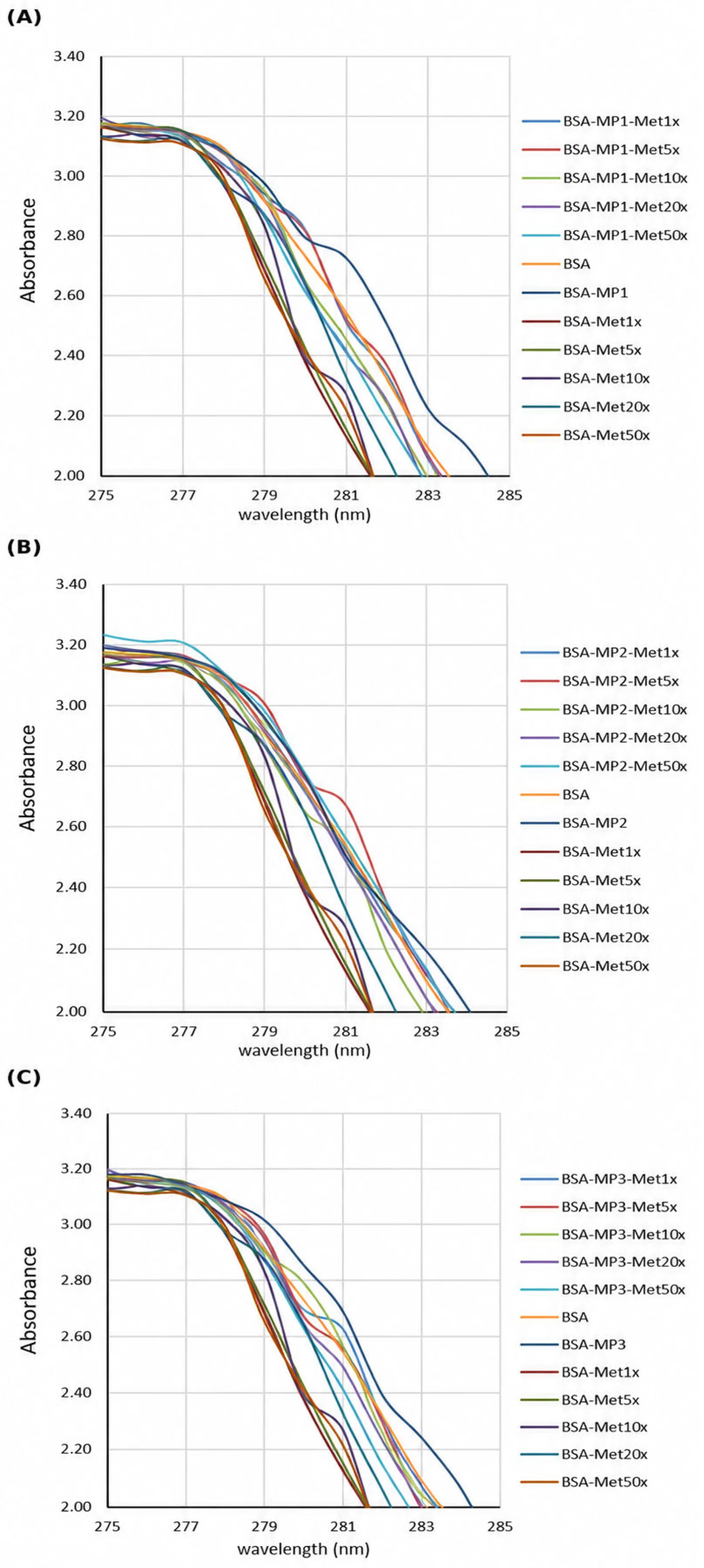
UV-vis spectra for (**A**) MP1 set, (**B**) MP2 set, and (**C**) MP3 set.

Figure 4 shows the changes in absorbance of BSA at 280 nm interacting with Met and PET MNPs. The absorbance value increases with complex formation. In the MP1 set, it was determined that the absorbance increased at low metformin concentrations (Met1x and Met5x) compared to BSA, while the absorbance value decreased with increasing concentration (Met10x, Met20x and Met50x) (Figure 4A). The difference between the absorbance values decreased with increasing plastic concentration. For the MP2 set, absorbance was lower at Met1x, Met10x and Met20x concentrations compared to BSA; and higher absorbance values were observed at Met5x and Met50x (Figure 4B). In the MP3 set, all concentrations except Met10x have lower absorbance values than BSA (Figure 4C).

### 2.3. Stability Indicators in Post-Filtration BSA–Metformin Liquid Phase

DLS and zeta-potential measurements showed formulation-dependent differences in the recovered particle-depleted fractions (Table 1). BSA alone exhibited a hydrodynamic size of 15.90 nm and a zeta potential of −15.6 mV. Following pre-incubation with PET MNPs, the hydrodynamic sizes of the BSA-containing fractions increased slightly to 16.81, 19.66, and 19.56 nm for BSA–MP1, BSA–MP2, and BSA–MP3, respectively. Correspondingly, their zeta potentials shifted toward less negative values of −7.31, −6.97, and −5.45 mV.

Metformin solutions displayed a broader variation in hydrodynamic size. The measured sizes were 8.60, 63.90, 33.60, 30.70, and 210.1 nm for Met1x, Met5x, Met10x, Met20x, and Met50x, respectively. In contrast, the zeta potential became progressively more negative with increasing metformin concentration, ranging from −8.9 mV for Met1x to −16.4 mV for Met50x.

The ternary BSA–MP–Met formulations exhibited hydrodynamic sizes ranging from 12.52 to 44.45 nm. In the MP1 series, the largest sizes were observed for BSA–MP1–Met1x (37.44 nm) and BSA–MP1–Met5x (44.45 nm), followed by lower values of 17.14–23.04 nm at the higher metformin levels. The MP2-containing formulations showed sizes between 12.52 and 29.52 nm, whereas the MP3-containing formulations ranged from 14.24 to 38.95 nm. The zeta potentials of the ternary systems remained negative and generally ranged from approximately −4.85 to −11.2 mV. No monotonic relationship between metformin concentration and either hydrodynamic size or zeta potential was observed in the ternary formulations.

### 2.4. Oxidative Indicators in Post-Filtration BSA–Metformin Liquid Phases

**Figure 5:**
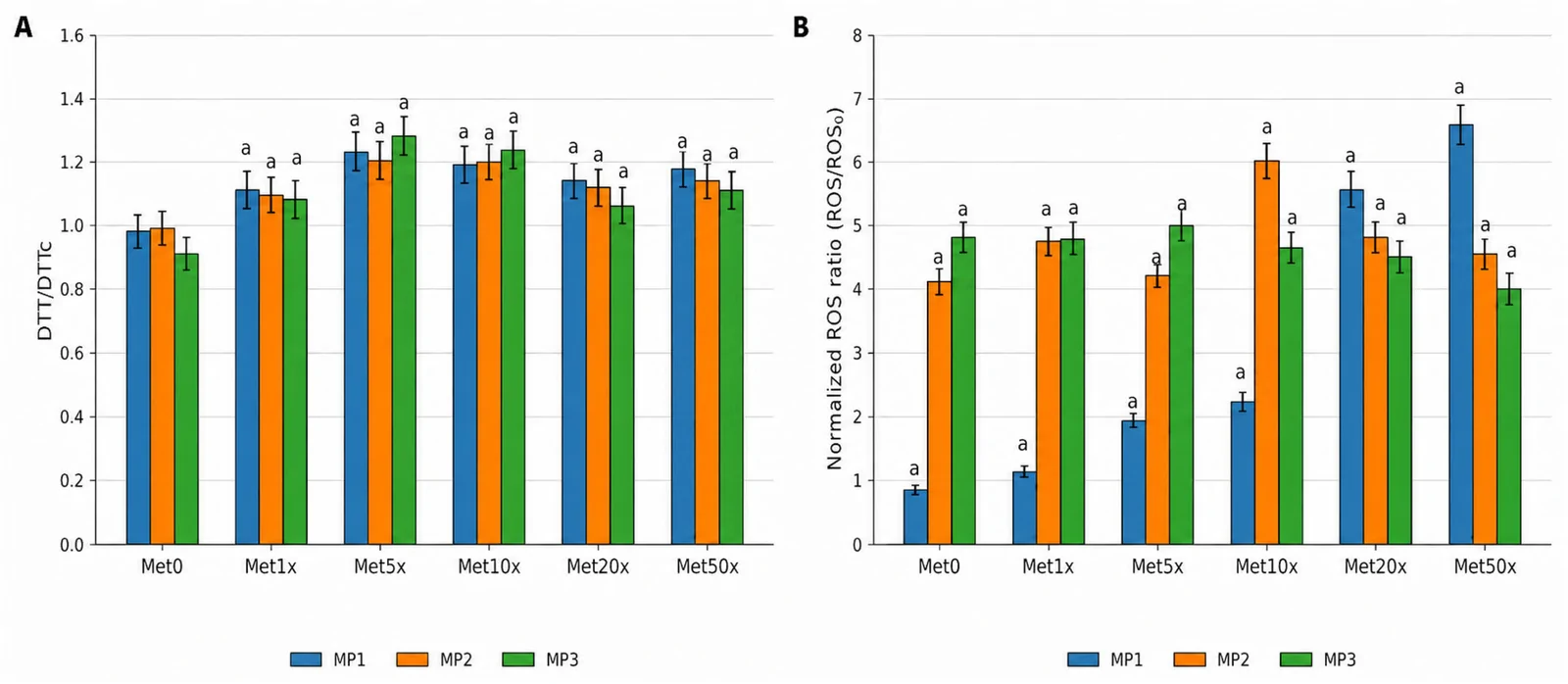
Oxidative indicators of the BSA–metformin system in the presence of PET MNPs. (**A**) DTT oxidative potential response expressed as DTT/DTTc ratio. (**B**) Normalized ROS response expressed as ROS/ROS_0_ ratio. Samples were evaluated at increasing metformin concentrations from Met0 to Met50x under three PET MNP loads: MP1, MP2, and MP3. Data are presented as mean ± SD; error bars indicate the standard deviation. DTT: DTT consumption measured in each experimental sample; DTTc: DTT consumption measured in the control sample without PET MNPs; ROS: reactive oxygen species level measured in each experimental sample; ROS_0_: ROS level measured in the control sample without PET MNPs. a: significantly different from control BSA and/or the corresponding group without MP (*p* < 0.05).

DTT analysis showed that normalized OP values increased after metformin exposure in the BSA–MET system with PET MNPs (Figure 5A). The Met0 control showed the lowest value, especially for MP3. At Met1x, all MP groups increased to a similar level. The highest OP response was observed at Met5x, with MP3 showing the greatest value, followed by MP1 and MP2. At Met10x, values remained elevated, again with MP3 slightly higher than MP1 and MP2. At Met20x and Met50x, OP values decreased slightly compared with Met5x/Met10x but remained above Met0 control. Overall, the DTT response followed a moderate dose-dependent increase up to Met5x–Met10x, with a partial decline at higher metformin levels.

Normalized ROS values showed distinct concentration-dependent patterns among the MP sets (Figure 5B). In the MP1 set, ROS increased progressively from Met0 to Met50x, rising from approximately 0.8 to 6.6. In the MP2 set, ROS remained elevated across all concentrations, with the highest value observed at Met10x, approximately 6.0. In the MP3 set, ROS was high at Met0–Met5x, approximately 4.8–5.0, followed by a gradual decrease at Met10x, Met20x, and Met50x, reaching approximately 4.0 at the highest concentration. At Met20x and Met50x, MP1 produced the highest ROS response among the three MP sets.

### 2.5. Responses of E. coli and S. aureus to Filtrates from PET-Conditioned BSA–Metformin Systems

**Figure 6:**
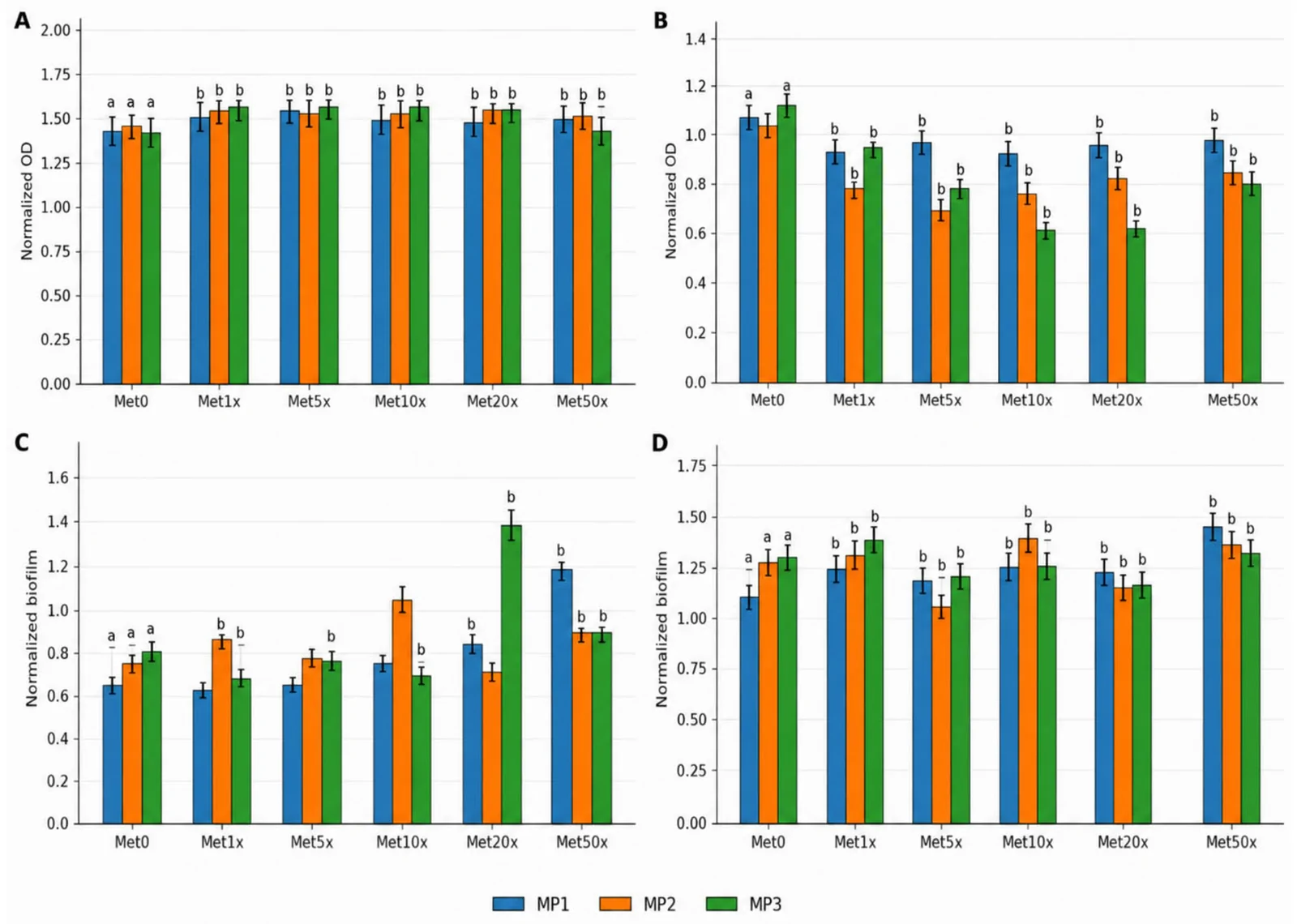
Bacterial growth (Optical density, OD) and biofilm formation of *E. coli* and *S. aureus* exposed to filtrates generated from PET-conditioned BSA–metformin systems. (**A**) OD of *E. coli* normalized to the bacterial control; (**B**) OD of *S. aureus* normalized to the bacterial control; (**C**) biofilm formation of *E. coli* normalized to the bacterial control. (**D**) Biofilm formation of *S. aureus* normalized to the bacterial control; MP1: 0.5 mg/mL, MP2: 2.5 mg/mL, MP3: 7.5 mg/mL PET MNPs. Met1x–Met50x indicate increasing metformin concentrations. Data are presented as mean ± SD; error bars indicate the standard deviation. Letters above bars indicate a: significantly different from control bacteria; b: significantly different from the corresponding group without MP (*p* < 0.05).

To assess the biological properties of the recovered liquid phases, *E. coli* and *S. aureus* were exposed to post-filtration samples generated from BSA–metformin systems previously conditioned with different PET MNP loads. The bacteria were not directly exposed to PET MNPs. Bacterial growth, oxidative-stress-related endpoints, lipid peroxidation, and biofilm formation were evaluated to determine whether differences among the recovered filtrates were associated with species-specific bacterial responses. Bacterial growth was monitored by optical density measurements (Figure 6). In *E. coli*, OD values in the MP1 set remained close to the Met0 condition across all metformin concentrations, with a slight decrease at Met10x (Figure 6A). In the MP2 set, Met1x produced a modest increase relative to Met0, whereas higher concentrations showed a gradual reduction from this peak. In the MP3 set, the strongest OD response was observed at Met1x and Met5x, followed by a decrease at Met50x. Overall, low metformin concentrations mildly enhanced *E. coli* growth in the presence of PET MNPs, whereas higher concentrations attenuated this growth-stimulatory response rather than producing clear inhibition.

In *S. aureus*, bacterial growth was more strongly reduced after metformin addition than in *E. coli* (Figure 6B). In the MP1 set, normalized OD decreased from Met0 to Met1x and remained below the bacterial control across the metformin-treated groups, with partial recovery at Met50x. The MP2 set showed the lowest OD at Met5x, followed by recovery at higher metformin concentrations. The MP3 set showed the strongest suppression, with OD decreasing from Met0 to a minimum at Met10x, followed by partial recovery at Met50x. These results indicate that *S. aureus* growth was more sensitive to exposure to filtrates generated from PET-conditioned BSA–metformin systems, particularly under higher PET MNP loads, although the response was non-linear rather than strictly concentration-dependent.

Biofilm formation was assessed by crystal violet staining at 595 nm (Figure 6C,D). In *E. coli*, biofilm formation showed MNP-dependent patterns (Figure 6C). In the MP1 set, biofilm decreased slightly at Met1x and Met5x compared with Met0, then increased at Met10x, Met20x, and Met50x, with the highest value at Met50x. In the MP2 set, biofilm increased at Met1x but decreased at higher metformin concentrations, remaining below Met0 at Met20x and Met50x. In the MP3 set, biofilm formation increased from Met0 to Met20x and then decreased at Met50x. These results show that PET MNPs and metformin differentially modulated *E. coli* biofilm formation depending on both PET MNP load and metformin concentration.

In *S. aureus*, biofilm formation was elevated above the bacterial control in all PET MNP sets and metformin concentrations (Figure 6D). In the MP1 set, biofilm remained consistently elevated, with the highest value at Met50x. The MP2 set showed a non-monotonic response, with the maximum biofilm level at Met10x. In the MP3 set, biofilm levels also remained elevated, with the highest value at Met1x and a lower response at Met20x before partial recovery at Met50x. Compared with *E. coli*, *S. aureus* showed a more uniform biofilm-promoting response under exposure to filtrates generated from PET-conditioned BSA–metformin systems.

**Figure 7:**
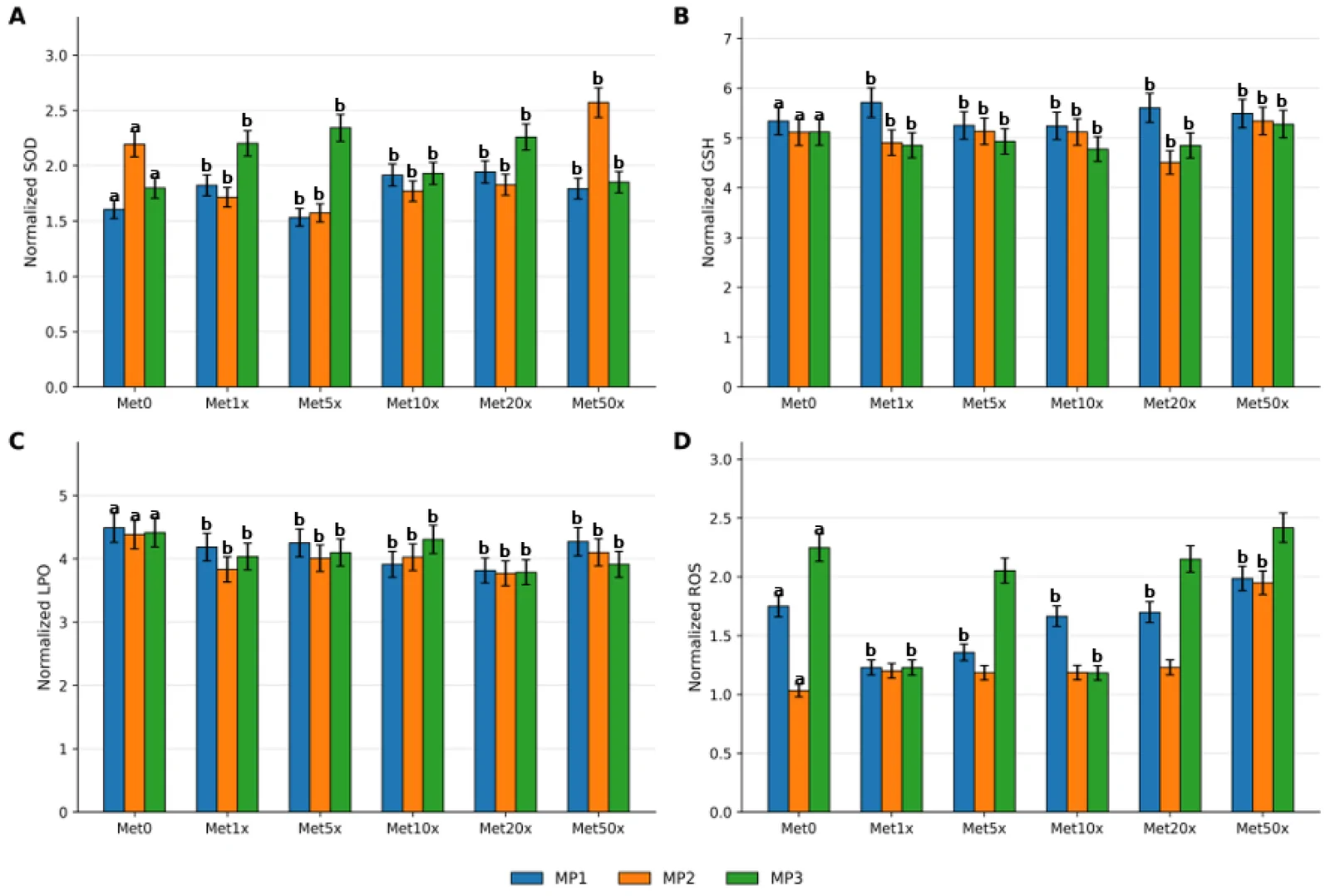
Oxidative stress markers in *E. coli* exposed to filtrates generated from PET-conditioned BSA–metformin systems: (**A**) SOD activity, (**B**) GSH activity, (**C**) LPO activity, and (**D**) ROS. All values were normalized to bacterial control. MP1: 0.5 mg/mL, MP2: 2.5 mg/mL, MP3: 7.5 mg/mL PET MNPs. Data are presented as mean ± SD; error bars indicate the standard deviation. Letters above bars indicate a: significantly different from control bacteria; b: significantly different from the corresponding group without MP (*p* < 0.05).

**Figure 8:**
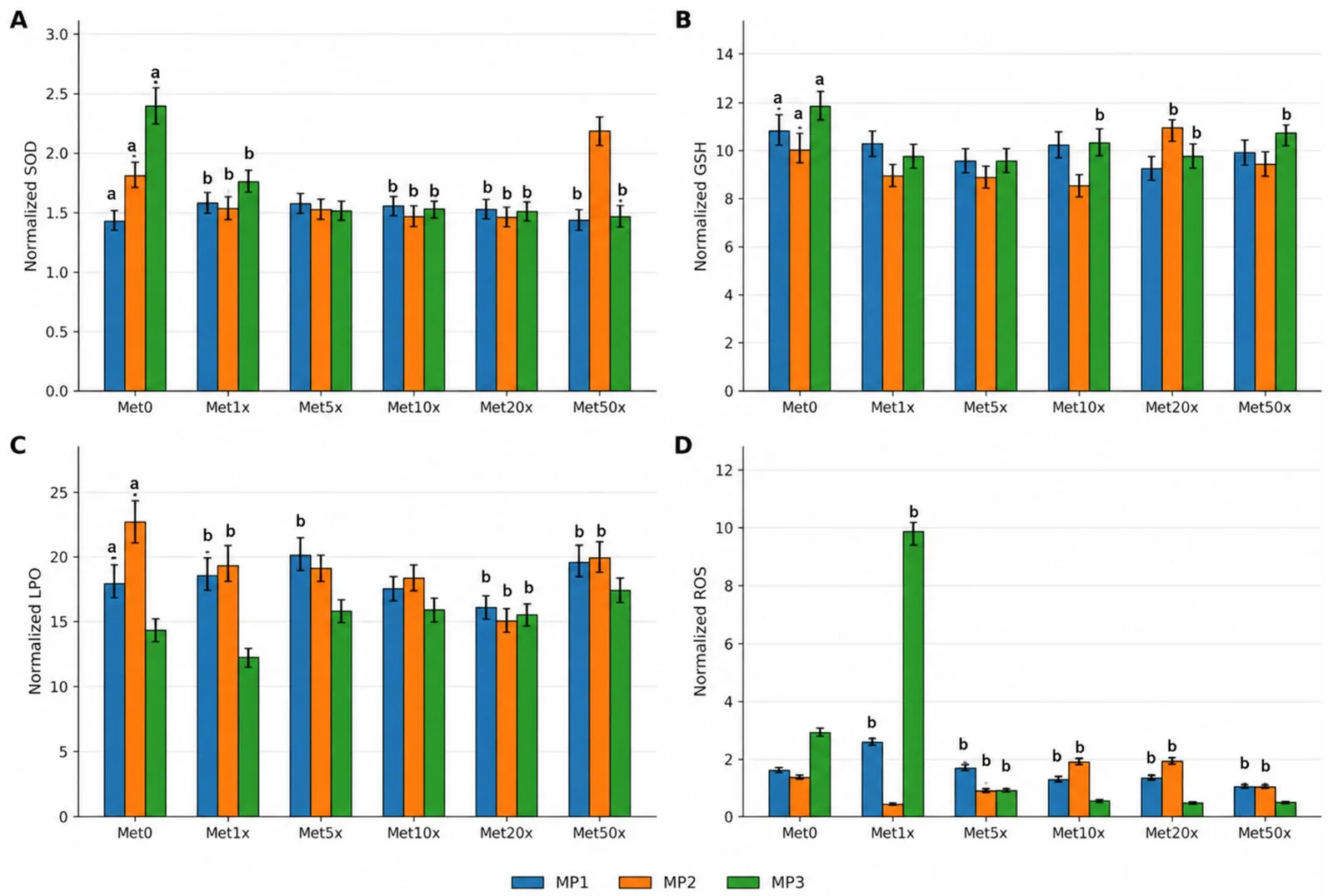
Oxidative stress markers in *S. aureus* exposed to filtrates generated from PET-conditioned BSA–metformin systems: (**A**) SOD activity, (**B**) GSH activity, (**C**) LPO activity, and (**D**) ROS. All values were normalized to bacterial control. MP1: 0.5 mg/mL, MP2: 2.5 mg/mL, MP3: 7.5 mg/mL PET MNP. Data are presented as mean ± SD; error bars indicate the standard deviation. Letters above bars indicate a: significantly different from control bacteria; b: significantly different from the corresponding group without MP (*p* < 0.05).

Cellular oxidative stress and antioxidant defense mechanisms were evaluated by measuring superoxide dismutase (SOD) activity, glutathione (GSH) content, reactive oxygen species (ROS) generation, and lipid peroxidation (LPO). As shown in Figure 7 and Figure 8, SOD activity served as a key indicator of the bacterial antioxidant response. In *E. coli*, normalized SOD activity varied across PET MNP sets and metformin concentrations (Figure 7A). In the MP1 set, SOD activity was highest at Met10x and Met20x. The MP2 set showed the strongest overall SOD induction, particularly at Met50x. In the MP3 set, the peak SOD response occurred at Met5x. These results indicate that PET MNP–metformin exposure induced a non-linear oxidative stress response in *E. coli*. In *S. aureus*, SOD activity was elevated above the bacterial control across all conditions (Figure 8A). In the MP1 set, SOD peaked at Met1x and then gradually decreased toward Met50x. In the MP2 set, SOD decreased from Met0 to Met20x, followed by a pronounced increase at Met50x. In the MP3 set, the highest SOD activity was observed at Met0, indicating that high PET MNP load alone induced a strong antioxidant response. Overall, *S. aureus* showed a robust but non-monotonic SOD response, with the strongest values observed in MP3-Met0 and MP2-Met50x.

GSH levels in *E. coli* showed relatively modest variation across conditions (Figure 7B). In the MP1 set, GSH ranged from approximately 5.24 to 5.71 μmol/g protein, with the highest value at Met1x. The MP2 set showed reduced GSH at Met1x and Met20x relative to Met0, while the MP3 set showed a decrease up to Met20x, followed by partial recovery at Met50x. Overall, GSH levels were largely maintained, suggesting that the GSH-based antioxidant pool was not severely depleted under the tested conditions. In *S. aureus*, GSH-related values remained relatively high across exposure groups (Figure 8B). The MP1 set ranged from 9.26 to 10.80 μmol/g protein, with the lowest value at Met20x and the highest at Met0. The MP2 set showed greater variability, with a decline at Met10x followed by a marked increase at Met20x. The MP3 set showed generally high values, ranging from 9.59 to 11.82 μmol/g protein. These results indicate that thiol-related antioxidant capacity was maintained in *S. aureus* despite oxidative stress induction.

LPO in *E. coli*, used as an index of oxidative membrane damage, decreased overall but non-monotonically with metformin addition (Figure 7C). LPO values were highest at Met0 in all MP sets and generally reached their lowest levels at Met20x, followed by partial recovery at Met50x. This pattern indicates that the addition of metformin was associated with reduced lipid peroxidation under several conditions, although the response was not strictly dose-dependent. In *S. aureus*, LPO showed a distinct pattern from *E. coli* and did not display a consistent declining trend with increasing metformin concentration (Figure 8C). In the MP1 set, LPO fluctuated across metformin concentrations, with the highest value at Met5x. In the MP2 set, LPO was highest at Met0, decreased to its lowest value at Met20x, and then increased again at Met50x. In the MP3 set, LPO values were lower overall and increased from Met1x toward Met50x, with some intermediate fluctuation. These findings indicate that lipid peroxidation responses were species-specific and strongly influenced by PET MNP load.

ROS levels were evaluated using normalized fluorescence measurements (Figure 7D and Figure 8D). In *E. coli*, the MP1 set showed the highest ROS signal at Met0, followed by a marked decrease after metformin addition. In the MP2 set, ROS remained relatively stable across Met1x–Met20x, with an increase at Met50x. In the MP3 set, ROS responses were more variable, with relatively higher values at Met0, Met5x, Met20x, and Met50x. These results indicate that PET MNPs and metformin modulated ROS generation in *E. coli* in an MP-load- and concentration-dependent manner. In *S. aureus*, ROS levels were highly variable, particularly in the MP3 set. In the MP1 set, ROS increased from Met0 to Met1x and then declined progressively toward Met50x. In the MP2 set, ROS decreased sharply at Met1x, increased again at Met10x and Met20x, and returned near baseline at Met50x. The most pronounced response was observed in the MP3 set, where ROS sharply increased at Met1x and then decreased markedly from Met5x onward. This indicates a transient and condition-specific oxidative response under high PET MNP load combined with low metformin concentration.

Comparative analysis via ANOVA for the bacterial responses showed that significant differences in growth were observed between *E. coli* and *S. aureus* response under exposure to filtrates generated from PET-conditioned BSA–metformin systems (*p* < 0.05). *E. coli* showed significant growth stimulation at low metformin concentrations, particularly under MP2 and MP3 conditions, whereas *S. aureus* showed significant growth suppression after metformin addition, especially under higher PET MNP load.

Biofilm formation differed significantly between the two bacterial species (*p* < 0.05). *E. coli* biofilm responses were MP-load dependent and non-monotonic, whereas *S. aureus* biofilm formation remained significantly elevated across most exposure groups compared with the bacterial control, indicating a stronger and more consistent biofilm-promoting response.

Oxidative stress markers showed significant exposure-dependent changes in both bacterial models (*p* < 0.05). In *E. coli*, SOD activity was significantly elevated under combined PET MNP–metformin exposure, while GSH levels remained comparatively stable, suggesting activation of antioxidant defense without severe thiol depletion. In *S. aureus*, SOD activity was significantly elevated across all exposure groups, with the strongest response observed under high PET MNP load, indicating greater sensitivity to PET MNP-associated oxidative stress.

LPO responses differed significantly between *E. coli* and *S. aureus* (*p* < 0.05). In *E. coli*, metformin addition significantly reduced LPO under several intermediate exposure conditions, whereas *S. aureus* showed fluctuating LPO responses without a consistent decreasing trend. These results indicate species-specific membrane oxidative damage patterns.

ROS production in bacteria showed significant species- and condition-specific differences (*p* < 0.05). The most pronounced ROS increase was observed in *S. aureus* under the MP3–Met1x condition, indicating a significant transient oxidative burst under high PET MNP load combined with low metformin concentration. This response was not observed in *E. coli*, supporting organism-specific oxidative sensitivity.

Metformin-only baseline responses were additionally evaluated to distinguish direct metformin effects from those observed in the BSA–PET–metformin-derived filtrates (Supplementary Figure S1). In *E. coli*, metformin alone produced only modest changes in bacterial growth (OD600), ROS, SOD, and GSH, while LPO and biofilm formation showed greater concentration-dependent variation. In *S. aureus*, metformin alone produced a different profile, characterized by increased OD600 and ROS, reduced SOD activity, relatively stable GSH-related responses, increased LPO, and variable biofilm formation. Thus, metformin alone generated species-specific baseline responses that differed from several of the patterns observed for the PET-conditioned BSA–metformin filtrates.

Overall, the bacterial results demonstrate that exposure to filtrates generated from PET-conditioned BSA–metformin systems produced species-specific biological responses. *E. coli* mainly showed growth stimulation with non-linear oxidative and biofilm responses, whereas *S. aureus* showed stronger growth suppression, consistently elevated biofilm formation, and a marked transient ROS increase under the MP3-Met1x condition.

## 3. Discussion

### 3.1. PET MNP-Mediated Modulation of BSA–Metformin Binding and Oxidative Indicators

The results obtained in this study indicate that the previously reported weak-to-moderate interaction between metformin and BSA is markedly altered in the presence of PET MNPs [18,19,20,21,22]. This finding supports the view that binding behavior observed in single-component systems may change under cocontamination conditions, where proteins, pharmaceuticals, and MNPs coexist and compete for interaction sites [18,19,20,26].

Synchronous fluorescence spectroscopy showed changes in the microenvironment of BSA aromatic residues, particularly Tyr and Trp, indicating local conformational rearrangements around these residues [35,36]. These changes suggest that metformin and PET MNPs modified the polarity, accessibility, or packing of the aromatic side-chain regions of BSA. Since Tyr and Trp residues are sensitive indicators of protein conformational changes, the observed spectral variations point to alterations in BSA stability and ligand accessibility. The fluorescence quenching behavior may be consistent with complex formation and possible static quenching; however, confirmation of the quenching mechanism would require additional lifetime or temperature-dependent fluorescence measurements.

UV–vis spectroscopy further supported changes in the aromatic amino acid environment of BSA. The absorption band around 280 nm is mainly associated with Trp, Tyr, and phenylalanine (Phe) residues [22]. Changes in this region therefore indicate alterations in the aromatic and hydrophobic microdomains of the protein. The increase in absorbance observed at some low metformin concentrations may reflect local rearrangement or complex formation in the aromatic residue environment, whereas the decrease at higher concentrations may be associated with conformational perturbation, shielding of aromatic residues, or aggregation-related optical effects. The modulation of these responses by PET MNPs suggests that the particles act not only as passive carriers but also as active interfacial surfaces that can shift the BSA–metformin binding equilibrium.

Rayleigh scattering and turbidity measurements provided complementary evidence for changes in aggregation behavior. Differences in Rayleigh scattering and turbidity indicate changes in the amount, size distribution, or scattering efficiency of filtration-passing soluble or colloidal species. Because the measurements were performed after 0.22 µm filtration, these results do not directly characterize the original PET MNP-containing suspension or larger complexes retained by the membrane. The observed signals may instead reflect smaller protein assemblies, filtration-passing colloids, differences in recovered protein concentration, or soluble constituents remaining in the analyzed phase [26,37]. In protein systems, such increases are commonly related to partial unfolding, exposure of interaction-prone regions, and aggregate formation [38]. In the present MNP–BSA–metformin ternary system, the concentration-dependent changes in both parameters suggest that metformin and PET MNPs jointly influence the aggregation tendency of BSA. PET MNPs may provide additional adsorption surfaces for both BSA and metformin, thereby promoting bridging interactions under some conditions. Conversely, at other concentrations, surface adsorption or protein stabilization may reduce protein–protein contact. Therefore, the Rayleigh scattering and turbidity results reflect competitive, concentration-dependent aggregation dynamics in the ternary system.

The DLS and zeta-potential results indicate that prior exposure to PET MNPs modified the physicochemical state of the recovered BSA–metformin fractions. Compared with BSA alone, the BSA–MP formulations showed a modest increase in hydrodynamic size accompanied by a pronounced shift toward less negative zeta potentials [39]. This decrease in the absolute surface charge suggests partial charge screening or modification of the electrostatic environment of the BSA-containing colloidal fraction after interaction with PET MNPs. Because the absolute zeta-potential values were generally well below the magnitude commonly associated with strong electrostatic stabilization, the recovered systems appear to possess relatively limited electrostatic colloidal stability [40].

The metformin-only samples showed a different pattern. Although the zeta potential became progressively more negative with increasing metformin concentration, the corresponding hydrodynamic sizes changed non-monotonically, with particularly high values at Met5x and Met50x. These findings indicate that the DLS response cannot be explained solely by increasing metformin concentration. The relatively large hydrodynamic species detected at selected concentrations may instead reflect concentration-dependent association phenomena or contributions from other colloidal components present in the pharmaceutical formulation.

The ternary BSA–MP–Met systems also displayed strongly non-monotonic changes in hydrodynamic size. Several formulations, particularly BSA–MP1–Met1x, BSA–MP1–Met5x, and BSA–MP3–Met20x, showed larger hydrodynamic dimensions than BSA alone, whereas other formulations returned to values close to the BSA-containing controls. Together with the variable zeta-potential shifts, these results suggest formulation-dependent reorganization of the soluble and nanoscale colloidal species rather than progressive aggregation driven simply by increasing metformin concentration.

Importantly, the measured DLS sizes should not be interpreted as the dimensions of the original PET MNPs or as direct measurements of PET–BSA corona particles [41]. The original PET particles were approximately 300–500 nm and were largely removed by sedimentation followed by 0.22 µm filtration before DLS analysis. Accordingly, the measured hydrodynamic diameters most likely represent soluble protein species and nanoscale colloidal structures remaining in the bulk particle-depleted filtrates. Therefore, the combined changes in hydrodynamic size and zeta potential support the conclusion that prior PET MNP exposure altered the colloidal and electrostatic state of the recovered BSA–metformin system even after removal of the bulk PET particle fraction.

Bradford analysis also supported changes in the accessibility of BSA binding regions. The Coomassie Brilliant Blue G-250 dye used in this method binds mainly through interactions with basic, aromatic, and hydrophobic residues of proteins [42,43]. Since the BSA amount was kept constant in all samples, changes in absorbance at 595 nm are likely related to altered dye accessibility rather than differences in total protein concentration. In the MNP–BSA–metformin system, metformin binding and/or PET MNP adsorption may mask or expose dye-accessible regions of BSA, leading to variation in Bradford response. Thus, the Bradford results are consistent with conformational rearrangement and altered surface accessibility of BSA in the ternary system.

DTT and cell free ROS analyses revealed that the MNP–BSA–metformin system also showed changes in oxidative indicators. ROS measurements indicated increased reactive oxygen species generation or ROS-like oxidative activity in several experimental groups, while DTT depletion reflected increased oxidative potential and consumption of reducing capacity [44,45,46]. When evaluated together, these findings suggest a shift of the system toward a more oxidizing redox environment. This response may be associated with conformational changes on the BSA surface, altered exposure of redox-sensitive residues, and PET MNP-mediated surface interactions that influence charge distribution and molecular accessibility [10,37,47]. The concentration-dependent behavior of metformin further suggests that it may exert either stabilizing or destabilizing effects depending on its level and the amount of PET MNPs present, consistent with previous reports showing weak metformin–albumin binding and metformin-induced albumin conformational changes [19,22].

The observed non-monotonic responses likely reflect concentration-dependent transitions in the physicochemical state of the BSA–metformin–PET system rather than a simple dose–response relationship. Partial protein coverage at low-to-intermediate PET levels may promote corona heterogeneity and protein-mediated particle bridging, whereas increased surface availability, adsorption-site saturation, redistribution of BSA and metformin, aggregate sedimentation, and filtration of larger complexes may dominate at higher PET levels. These processes can nonlinearly alter the composition of the soluble and filter-passing fraction, as evidenced by the non-monotonic hydrodynamic size variations observed in our DLS analysis (Table 1).

The corresponding bacterial growth and ROS patterns may therefore arise from the balance between adaptive metabolic or antioxidant responses at moderate stress and oxidative, membrane, or protein-associated damage above a critical exposure threshold. Nevertheless, although DLS and zeta potential measurements confirm the colloidal reorganization in the recovered phases, the precise competitive adsorption affinities, real-time aggregation kinetics, and exact molecular speciation within the filtrates were not independently determined. Thus, these complex interaction pathways remain mechanistic hypotheses.

Overall, the combined fluorescence, UV–vis, Bradford, Rayleigh scattering, turbidity, DTT, and ROS results indicate that PET MNPs modulate metformin–BSA interactions through changes in aromatic residue microenvironments, protein surface accessibility, aggregation behavior, and oxidative potential. These findings suggest that the MNP–BSA–metformin ternary system is governed by competitive and concentration-sensitive interactions rather than by the independent effects of metformin or PET MNPs alone.

### 3.2. Bacterial Responses to PET-Conditioned BSA–Metformin Filtrates

The metformin-only controls provide additional context for distinguishing direct drug responses from those associated with the PET-conditioned BSA system. In *E. coli*, metformin alone did not reproduce the growth stimulation observed under several PET-conditioned BSA–metformin conditions, whereas in *S. aureus*, metformin alone tended to increase OD600 rather than reproduce the growth suppression observed in the ternary filtrates. Similarly, the metformin-only oxidative and biofilm profiles differed from several responses observed after PET conditioning. Together with the BSA+MP (Met0) controls included in the main experimental series, these findings suggest that the bacterial responses to the ternary filtrates cannot be attributed solely to the direct action of metformin, but instead reflect modulation of the recovered BSA–metformin phase following prior PET MNP exposure. Nevertheless, because a complete PET-only bacterial control series was not evaluated across all endpoints, the individual contribution of PET-derived soluble constituents cannot be fully separated.

The bacterial assays showed that post-filtration liquid phases generated from BSA–metformin systems conditioned with different PET loads produced concentration-dependent but non-linear responses in both *E. coli* and *S. aureus*. Because the visibly settled PET material was not resuspended and the upper phase was filtered before bacterial exposure, MP1, MP2, and MP3 represent upstream PET-conditioning conditions rather than direct exposure of bacterial cells to the original 300–500 nm particles. Accordingly, the observed effects are attributed primarily to differences in the soluble and filter-passing fractions, although the contribution of residual sub-220 nm fragments cannot be completely excluded.

The growth responses differed between the two species. In *E. coli*, OD values remained above the bacterial control across the tested filtrates, indicating no clear growth inhibition under the experimental conditions. Filtrates generated using MP2 and MP3 produced the highest OD responses at low-to-intermediate metformin concentrations, particularly around Met1x–Met5x, whereas this stimulation was attenuated at higher concentrations. In contrast, metformin-containing filtrates generally reduced *S. aureus* growth, especially under the MP3 condition, with the lowest response observed near Met10x followed by partial recovery at Met50x. These findings indicate that the biological properties of the recovered liquid phases depended on both the initial PET load and metformin concentration and that the resulting responses were species-specific. Metformin has previously been reported to influence bacterial growth, membrane-associated processes, and biofilm-related phenotypes in a concentration-dependent manner [48,49,50,51].

Both organisms displayed evidence of oxidative stress, although the response patterns differed. SOD activity increased across most exposure conditions, indicating an enhanced antioxidant response to superoxide-associated stress [52,53]. In *E. coli*, the strongest SOD responses occurred under combined PET-conditioning and metformin conditions, particularly MP2–Met50x and MP3–Met5x. In *S. aureus*, the highest SOD activity was observed in MP3–Met0, suggesting that the filtrate generated under the highest PET-conditioning load was sufficient to induce a strong oxidative response even without metformin. The thiol-reactive antioxidant signal remained comparatively stable in both species, suggesting that low-molecular-weight thiol buffering was not completely exhausted. For *S. aureus*, this response should be interpreted as general thiol-based antioxidant capacity because bacillithiol, rather than glutathione, is its principal low-molecular-weight thiol [54,55].

Lipid peroxidation and ROS responses were also non-monotonic. In *E. coli*, LPO generally decreased at intermediate metformin concentrations but increased again under some high-concentration conditions. This pattern should not be interpreted as direct membrane protection by metformin because extracellular DTT and ROS measurements characterize the oxidative properties of the filtrates, whereas bacterial LPO reflects the integrated cellular response, including growth status, uptake, antioxidant-enzyme activity, and membrane-specific stress. Increased SOD activity may have limited lipid oxidation under some conditions, but this was not directly tested. In *S. aureus*, LPO fluctuated without a consistent directional trend, indicating a different membrane response to the recovered filtrates.

The most pronounced ROS response occurred in *S. aureus* under MP3–Met1x, where a sharp but concentration-specific increase was observed. ROS declined at higher metformin concentrations, indicating that this effect did not follow a simple dose-dependent relationship. *E. coli* showed more moderate and PET-load-dependent ROS changes. These non-linear profiles may reflect changes in protein conformation, competitive adsorption of BSA and metformin, heterogeneous aggregate formation, sedimentation, and selective removal of larger complexes during filtration. Consistent with this interpretation, DLS and zeta-potential measurements of the recovered filtrates demonstrated formulation-dependent and non-monotonic changes in hydrodynamic size and surface charge, supporting reorganization of the soluble and nanoscale colloidal fraction. Such physicochemical changes could alter the composition and bioavailability of the filter-passing phase without producing a proportional relationship with the initial PET load. However, the underlying mechanisms remain hypothetical because competitive adsorption, aggregation kinetics, molecular speciation, and the PET-specific identity and size distribution of residual filter-passing particles or colloids were not independently resolved.

Biofilm formation further distinguished the two organisms. The *E. coli* response was variable, with limited changes at lower metformin concentrations and stronger stimulation under some higher-concentration conditions. In contrast, *S. aureus* biofilm formation remained above the bacterial control across all tested filtrates, with marked responses in MP1–Met50x, MP2–Met10x, and MP3–Met1x. These findings suggest that *S. aureus* more consistently shifted toward a biofilm-associated stress phenotype. Oxidative stress and changes in extracellular chemical composition are known to influence bacterial attachment, matrix production, and stress tolerance [56,57,58,59,60].

The contrasting responses may partly reflect differences in cell-envelope architecture. The lipopolysaccharide-containing outer membrane of *E. coli* provides an additional selective barrier that can limit access of xenobiotics, protein-associated compounds, and other filter-passing constituents to the cytoplasmic membrane. In comparison, *S. aureus* lacks an outer membrane and possesses a thick but porous peptidoglycan layer containing negatively charged teichoic acids, which may favor interactions with charged or protein-associated components. Differences between glutathione-related defenses in *E. coli* and bacillithiol-dependent buffering in *S. aureus* may also contribute to their distinct oxidative profiles. Nevertheless, permeability, surface binding, and intracellular uptake were not directly measured, and these explanations should therefore be regarded as plausible rather than confirmed mechanisms.

Overall, upstream PET conditioning altered the biological properties of BSA–metformin filtrates in a species- and concentration-dependent manner. *E. coli* mainly exhibited adaptive and non-linear changes in growth, oxidative-stress markers, lipid peroxidation, and biofilm formation, whereas *S. aureus* showed stronger growth suppression, a marked condition-specific ROS increase, and more consistent biofilm stimulation. These findings describe responses to the soluble and filter-passing fraction generated by the PET–BSA–metformin system and should not be interpreted as evidence of direct interaction between the original PET particles and bacterial cells. Translation of these findings to in vivo systems should therefore be approached cautiously. In vivo, dynamic protein-corona evolution, tissue distribution, cellular and immune uptake, metabolism, and renal or hepatobiliary clearance may continuously alter the composition, persistence, and bioavailability of PET-associated soluble and nanoscale colloidal species.

**Figure 9:**
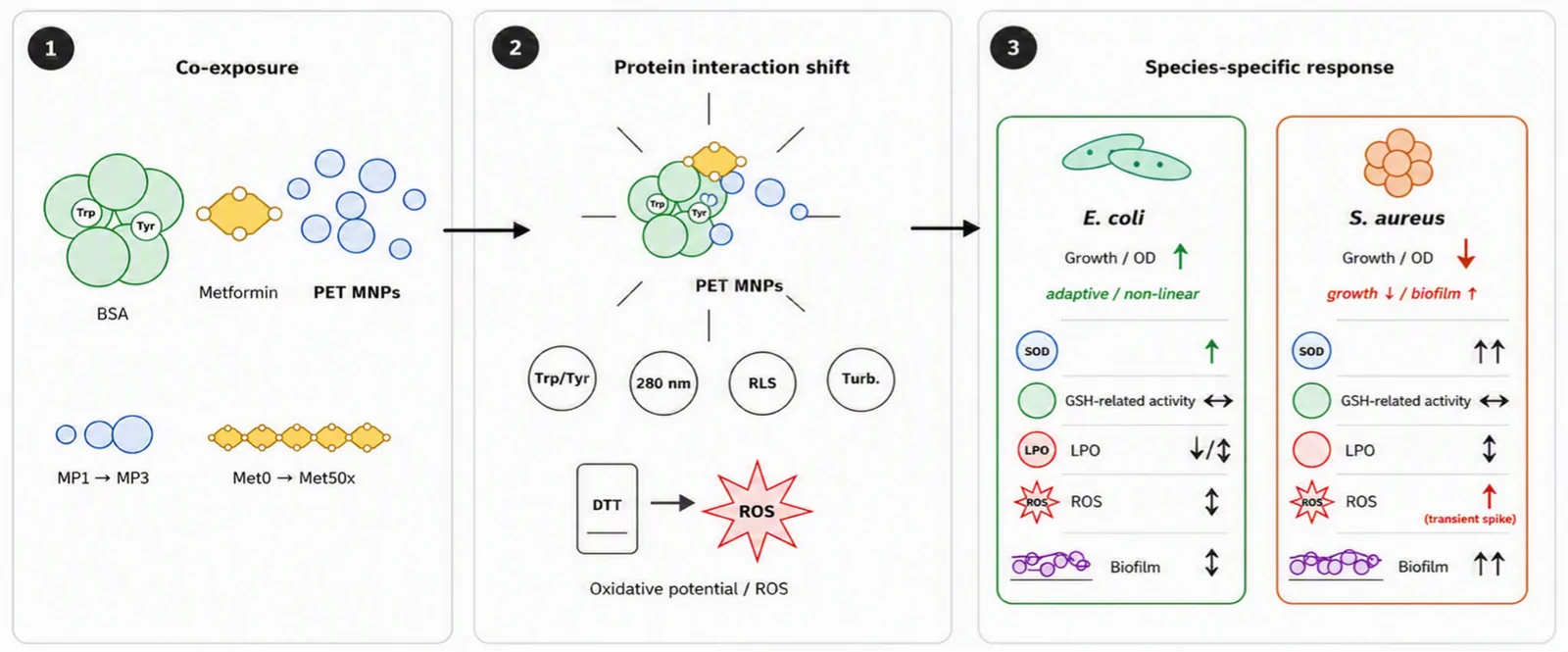
Conceptual summary of PET MNP-mediated changes in BSA–metformin interactions and the species-dependent bacterial responses induced by the resulting particle-depleted filtrates. The proposed interaction pathways are interpretative and do not represent directly confirmed molecular mechanisms.

As summarized in Figure 9, PET MNP conditioning altered the physicochemical characteristics of the BSA–metformin system and generated non-linear, species-dependent biological responses. Fluorescence and UV–Vis measurements indicated changes in the Trp and Tyr microenvironments of BSA, while Bradford, Rayleigh scattering, and turbidity analyses suggested alterations in protein surface accessibility and aggregation state. DTT and ROS assays further showed that the oxidative characteristics of the ternary system varied with PET load and metformin concentration. The resulting particle-depleted filtrates produced predominantly adaptive and non-linear responses in *E. coli*, whereas *S. aureus* exhibited stronger growth suppression, a condition-specific ROS increase, and more consistent biofilm stimulation. Because binding thermodynamics, molecular speciation, and residual particles in the filtrates were not directly determined, Figure 9 represents a conceptual summary of the observed trends rather than a confirmed molecular mechanism.

## 4. Materials and Methods

### 4.1. Preparation of PET MNPs

In this study, PET MNPs were generated by mechanically shredding commercially used PET water bottles with a stainless-steel grater, following a previously described in-house protocol [28,29]. The shredded material was then passed through a 100-mesh sieve, washed with pure water, and dried. This specific PET MNP batch had already been characterized by particle-size distribution/TEM (HT7700, Minato, Japan), EDX (SEM–EDX, FEI, Thermo Fisher Scientific, Waltham, MA, USA), BET sorption–desorption analysis (Micromeritics Gemini VII 2390 T, Norcross, GA, USA), FTIR (Bruker Invenio S, Billerica, MA, USA), XRD (PANalytical Xpert Pro X-ray Diffractometer, Malvern, UK), ^1^H-NMR (Agilent 500MHz, Santa Clara, CA, USA), and zeta-potential (DLS, Zetasizer Nano ZS, Malvern Instruments, Malvern, UK) measurements (Supplementary Figure S2). These reported physicochemical, structural, and surface-characterization data supported the classification of the material as PET MNPs [28,29]. The generated PET MNPs exhibited an average particle size of 374 ± 56 nm, with the overall size distribution tightly constrained between 250 nm and 490 nm, as confirmed by the size distribution profile (Supplementary Figure S2b).

### 4.2. Exposure Procedure of PET MNPs with BSA and Met

The PET MNPs were added to the exposure mixtures at final concentrations of 0.5, 2.5, and 7.5 mg/mL, designated MP1, MP2, and MP3, respectively. These concentrations were obtained by adding 0.002, 0.01, or 0.03 g of PET MNPs to a final mixture volume of 4 mL. Bovine serum albumin (BSA; GoldBio, St. Louis, MO, USA) was initially prepared at 4 mg/mL. Metformin (Met) solutions, prepared from a commercially available pharmaceutical formulation, were initially prepared at 0.2 µg/mL (Met1x), 1 µg/mL (Met5x), 2 µg/mL (Met10x), 4 µg/mL (Met20x), and 10 µg/mL (Met50x). For each exposure mixture, 1 mL of the corresponding metformin solution was combined with 3 mL of BSA solution in the presence of the specified PET MNP concentration, resulting in a final BSA concentration of 3 mg/mL and final metformin concentrations of 0.05, 0.25, 0.50, 1.0, and 2.5 µg/mL, respectively. The mixtures were incubated at 37 °C for 1 h. Following incubation, PET-containing material that had visibly settled or accumulated at the bottom of the tubes was not resuspended. The upper aqueous phase was carefully aspirated using a pipette without disturbing the sediment and was subsequently passed through a 0.22 µm low-protein-binding syringe filter. Thus, syringe filtration was used as a final clarification step rather than as the primary method for removal of the original 300–500 nm PET particles.

To evaluate potential filtration-related losses of BSA and metformin, recovery experiments were performed at three representative concentration levels spanning the low, intermediate, and high ranges of the experimental concentrations. Calibration curves were established separately for BSA and metformin, and concentrations were determined before and after passage through the 0.22 µm low-protein-binding syringe filter. Comparative analysis showed no statistically significant differences between pre- and post-filtration measurements at the tested concentration levels (*p* > 0.05), indicating that filtration-related retention of soluble BSA and metformin was not significant under the applied experimental conditions.

BSA-only, metformin-only, and BSA–metformin control solutions were subjected to the same transfer and filtration procedure as the PET-containing formulations. Additional PET-related control formulations were likewise processed under identical experimental conditions, thereby standardizing potential effects associated with tube handling, syringe contact, membrane filtration, and incubation across the experimental groups.

The resulting bulk particle-depleted filtrates were used for all subsequent chemical, spectroscopic, and bacterial analyses. This procedure was expected to remove the majority of the original 250–490 nm PET particle mass. However, passage of newly generated particles or fragments smaller than the nominal 220 nm filter pore size, as well as removal of large PET-associated protein aggregates during sedimentation or filtration, cannot be completely excluded. Accordingly, MP1, MP2, and MP3 denote the amounts of PET MNPs present during the pre-filtration incubation stage and should not be interpreted as residual particle concentrations in the final analytical samples. Because residual particle concentrations were not independently quantified after filtration, the recovered samples are referred to throughout the manuscript as bulk particle-depleted filtrates rather than particle-free supernatants.

### 4.3. Investigation of Protein Binding Properties After Treatment with PET MNP by Fluorescence and UV-Vis Spectroscopy

The ability of BSA to bind to PET MNPs in the presence of metformin was measured after filtration using fluorescence (Thermo Scientific VarioScan LUX Multimode microplate reader, Cleveland, OH, USA) and UV-Vis spectrophotometers (Thermo Scientific MultiScan Skyhigh microplate reader, Cleveland, OH, USA). All subsequent physicochemical, spectroscopic, oxidative, and bacterial analyses were performed on these recovered bulk particle-depleted filtrates. Fluorescence spectra of BSA alone and metformin alone were recorded at the same concentrations and exposure stages. Spectrum scanning was performed on BSA at an excitation wavelength of 280 nm in the emission wavelength range of 300–500 nm and at an excitation wavelength of 296 nm in the emission wavelength range of 300–600 nm, and it was determined with Δλ = 60 nm (Δλ = λemi − λexc, the difference between excitation and emission wavelengths). Fluorescence, UV–vis, and Bradford measurements were performed on the post-filtration liquid phases obtained from BSA–metformin systems previously conditioned with or without PET MNPs. PET MNPs were not intentionally present during these measurements. Consequently, the measured responses represent the recoverable soluble and filtration-passing fractions and may reflect changes in fluorophore microenvironment, molecular composition, protein recovery, or the presence of filtration-passing soluble or colloidal constituents.

Bradford analysis was applied to obtain information about the binding sites of BSA, and absorbance measurement after Coomassie Brilliant Blue G250 dye (Biobasic, Markham, ON, Canada) addition was performed at a wavelength of 595 nm.

### 4.4. Light-Scattering and Turbidity Properties of Post-Filtration BSA–Metformin Liquid Phases

Rayleigh scattering and turbidity measurements were performed on the filtrates generated from BSA–metformin systems previously conditioned with different PET MNP loads. Rayleigh scattering was measured at an excitation wavelength of 350 nm over the wavelength range of 300–400 nm, and turbidity was measured at 600 nm. Because PET MNPs and larger retained complexes were removed before analysis, these measurements describe light-scattering properties of the filtration-passing fraction and not direct scattering by the original PET MNP suspension.

### 4.5. Investigation of Aromatic Side Chain and Protein Folding Properties of BSA in the Presence and Absence of PET MNP

The absorbance of BSA and metformin solutions treated with and without PET MNP was scanned between 200–600 nm. Baselines of blank solutions were extracted from the sample absorbance. Absorbances around 280 nm were used to determine aromatic side chain and opened protein properties after MNP-met treatment.

### 4.6. DLS and Zeta Potential Measurements

The hydrodynamic size and zeta potential of filtered solutions with and without PET MNPs were measured using a Zetasizer instrument (Malvern Panalytical, Worcestershire, UK). Samples were equilibrated at room temperature before analysis, and measurements were performed in replicate. Results are reported as mean ± standard deviation.

### 4.7. Bacterial Exposure and Bacterial Assays

*E. coli* ATCC 35218 and *S. aureus* ATCC 25923 were incubated for 24 h with aliquots of the same post-filtration liquid phases used for the chemical and spectroscopic analyses. These filtrates were obtained from BSA–metformin systems previously conditioned with or without PET MNPs. PET MNPs were not directly added to the bacterial cultures. Therefore, bacterial responses were evaluated as effects associated with the composition of the recovered filtrates rather than as consequences of direct bacterial contact with PET particles [28,61]. A bacterial control without BSA-drug systems exposure was also included. Consequently, the assay was designed to compare growth and stress-related endpoints, not to assess antibiotic susceptibility or changes in resistance.

Bacterial growth was estimated by optical density at 600 nm UV-Vis spectrometer (Thermo Scientific VarioScan LUX Multimode microplate reader, Cleveland, OH, USA). OD600 is a turbidity-based growth proxy and was not interpreted as a direct viability or colony-count measurement. Following the OD600 measurements for bacterial growth, the same bacterial samples and controls were used for oxidative indicators. Biofilm formation was assessed using Crystal violet assay dye (Biobasic, Markham, ON, Canada). Intracellular ROS was measured using DCFH-DA at 488/530 nm. SOD activity was evaluated using the nitroblue tetrazolium (Biobasic, Markham, ON, Canada) method at 560 nm [29,61]. GSH-related activity was measured according to Arora et al. (2008) [62] using sodium azide (Biobasic, Markham, ON, Canada), glutathione reductase (Biobasic, Markham, ON, Canada), NADPH (Biobasic, Markham, ON, Canada), and H_2_O_2_ (Merck, Darmstadt, Germany), with absorbance monitored at 340 nm. LPO was measured using the TBARS assay at 532 nm [29,61,63].

### 4.8. Statistical Analysis

Data are presented as mean ± standard deviation (SD) from independent experiments. Differences among experimental groups were evaluated using one-way analysis of variance (ANOVA), followed by Tukey’s multiple-comparison test. For physicochemical assays, PET-conditioned formulations were compared with the corresponding PET-free formulation at the same metformin concentration. For bacterial assays, two predefined comparisons were evaluated: (a) versus the untreated bacterial control and (b) versus the corresponding BSA–metformin formulation without prior PET MNP conditioning. Differences were considered statistically significant at *p* < 0.05.

## 5. Conclusions

This study showed that conditioning the BSA–metformin system with commercial water-bottle-derived PET MNPs produced concentration-dependent changes in protein-associated spectroscopic, colloidal, and oxidative characteristics. Fluorescence and UV–Vis measurements indicated perturbations in the Trp and Tyr microenvironments of BSA, while Bradford, Rayleigh scattering, and turbidity analyses suggested changes in protein accessibility and aggregation state. DLS and zeta-potential measurements further demonstrated formulation-dependent changes in the hydrodynamic size and electrostatic properties of the recovered bulk particle-depleted fractions, supporting reorganization of the soluble and nanoscale colloidal species following PET MNP conditioning. DTT and ROS assays also showed that the oxidative characteristics of the recovered liquid phase varied according to the initial PET load and metformin concentration.

The resulting bulk particle-depleted filtrates produced non-linear and species-dependent bacterial responses. *E. coli* predominantly exhibited adaptive changes, including growth stimulation, variable oxidative-stress responses, and condition-dependent biofilm formation. In contrast, *S. aureus* showed greater growth suppression following metformin exposure, consistently elevated biofilm formation, and a pronounced ROS increase under the MP3–Met1x condition. Differences in cell-envelope structure and antioxidant regulation may contribute to these contrasting responses, although direct mechanistic confirmation is required.

Several limitations should be considered. The experimental design did not distinguish the effects of PET-derived soluble constituents and leached additives from other changes generated during PET–BSA–metformin conditioning. Although DLS and zeta-potential measurements characterized the hydrodynamic and electrostatic properties of the post-filtration fractions, these measurements could not distinguish residual sub-220 nm PET fragments from protein- or protein–metformin-associated colloidal structures, and the residual PET-specific particle concentration was not independently quantified. In addition, although filtration-recovery experiments indicated negligible loss of soluble BSA and metformin, retention of larger BSA aggregates or PET-associated protein complexes during sedimentation and filtration could not be independently quantified. Fluorescence-lifetime and multi-temperature measurements were also not performed; therefore, the quenching mechanism, binding affinity, binding-site number, and thermodynamic parameters could not be established. Moreover, translation of these findings to in vivo systems should be made cautiously because protein-corona evolution, tissue distribution, metabolism, cellular uptake, and physiological clearance may substantially modify the exposure profile.

Overall, the findings indicate that upstream PET MNP conditioning altered the physicochemical state and biological activity of the filtration-passing BSA–metformin phase. The results should therefore be interpreted as integrated effects of the recovered soluble and nanoscale colloidal fraction rather than as evidence of direct interaction between the original PET particles and bacterial cells.

## Figures and Tables

**Table 1:** Hydrodynamic size and zeta potential of BSA, metformin, BSA–PET MNP, and BSA–PET MNP–metformin formulations measured in the recovered bulk particle-depleted fractions after sedimentation and 0.22 µm filtration (SD ˂ 3.7).

Sample	Zeta Potentials, mV	Size, nm
BSA	−15.6	15.90
MP1	−8.1	12.90
MP2	−5.4	6.70
MP3	−0.2	8.10
Met1x	−8.9	8.60
Met5x	−10.6	63.90
Met10x	−12.1	33.60
Met20x	−13.8	30.70
Met50x	−16.4	210.1
BSA-MP1	−7.31	16.81
BSA-MP2	−6.97	19.66
BSA-MP3	−5.45	19.56
BSA-MP1-Met1x	−7.73	37.44
BSA-MP1-Met5x	−8.64	44.45
BSA-MP1-Met10x	−9.04	17.14
BSA-MP1-Met20x	−8.24	20.3
BSA-MP1-Met50x	−9.7	23.04
BSA-MP2-Met1x	−4.85	16.7
BSA-MP2-Met5x	−9.93	18.20
BSA-MP2-Met10x	−7.22	12.52
BSA-MP2-Met20x	−5.98	29.52
BSA-MP2-Met50x	−11.2	21.75
BSA-MP3-Met1x	−8.82	30.15
BSA-MP3-Met5x	−5.99	20.09
BSA-MP3-Met10x	−10.7	18.72
BSA-MP3-Met20x	−7.31	38.95
BSA-MP3-Met50x	−5.61	14.24

## Data Availability

The original contributions presented in this study are included in the article. Further inquiries can be directed to the corresponding author(s).

## Supplementary Materials

The following supporting information can be downloaded at https://www.mdpi.com/article/10.3390/ijms27167504/s1.

## References

[1] Gigault J., Hadri H.E., Nguyen B., Grassl B., Rowenczyk L., Tufenkji N., Feng S., Wiesner M.R. (2021). Nanoplastics are neither microplastics nor engineered nanoparticles. Nat. Nanotechnol..

[2] Luo Q., Tan H., Ye M., Jho E.H., Wang P., Iqbal B., Zhao X., Shi H., Lu H., Li G. (2025). Microplastics as an emerging threat to human health: An overview of potential health impacts. J. Environ. Manag..

[3] Thompson R.C., Courtene-Jones W., Boucher J., Pahl S., Raubenheimer K., Koelmans A.A. (2024). Twenty years of microplastic pollution research—What have we learned?. Science.

[4] Chen H., Wang X., Yin H., Sun L., Yang X., Zhong W., Kong L., Liu L. (2025). Microplastic surface biofilms: A review of structural characteristics and environmental implications. Front. Mar. Sci..

[5] Wright S.L., Kelly J.F. (2017). Plastic and Human Health: A Micro Issue?. Environ. Sci. Technol..

[6] Vethaak A.D., Legler J. (2021). Microplastics and human health: Knowledge gaps should be addressed to ascertain the health risks of microplastics. Science.

[7] Wright S.L., Thompson R.C., Galloway T.S. (2013). The physical impacts of microplastics on marine organisms: A review. Environ. Pollut..

[8] Zhang J., Wang L., Kannan K. (2021). Quantitative analysis of polyethylene terephthalate and polycarbonate microplastics in sediment collected from South Korea, Japan and the USA. Chemosphere.

[9] Dmitrowicz A., Sacharczuk M., Skiba D. (2026). Micro- and nanoplastics in human biological materials: A systematic review of detection methods and methodological challenges. Arch. Toxicol..

[10] Rajendran D., Chandrasekaran N., Waychal Y., Mukherjee A. (2022). Nanoplastics alter the conformation and activity of human serum albumin. NanoImpact.

[11] Baysal A., Saygin H., Soyocak A., Kahraman M., Apaydin E., Ozbek P. (2025). Elucidating the leaching effect of micro-/nano-plastics on the binding, structural, and oxidative characteristics of bovine serum albumin and its impact on cytotoxicity and oxidative stress in the human lung cancer cell line A549. Environ. Sci. Nano.

[12] Peters T. (1995). All About Albumin: Biochemistry, Genetics, and Medical Applications.

[13] Bujacz A. (2012). Structures of bovine, equine and leporine serum albumin. Acta Crystallogr. Sect. D Biol. Crystallogr..

[14] Fasano M., Curry S., Terreno E., Galliano M., Fanali G., Narciso P., Notari S., Ascenzi P. (2005). The extraordinary ligand binding properties of human serum albumin. IUBMB Life.

[15] Belinskaia D.A., Voronina P.A., Batalova A.A., Goncharov N.V. (2020). Serum albumin. Encyclopedia.

[16] Du K., Du X., Sun Z., Wang Y., Bao Y., Lan J., Shi R., Zhao Z. (2024). Effects of polystyrene nanoplastics on the binding of ciprofloxacin to bovine serum albumin. Colloids Surf. A Physicochem. Eng. Asp..

[17] Petitpas I., Bhattacharya A.A., Twine S., East M., Curry S. (2001). Crystal structure analysis of warfarin binding to human serum albumin: Anatomy of drug site I. J. Biol. Chem..

[18] Rahnama E., Mahmoodian-Moghaddam M., Khorsand-Ahmadi S., Saberi M.R., Chamani J. (2014). Binding site identification of metformin to human serum albumin and glycated human serum albumin by spectroscopic and molecular modeling techniques: A comparison study. J. Biomol. Struct. Dyn..

[19] Chaves O.A., Mathew B., Parambi D.G.T., de Oliveira C.H.C.S., Cesarin-Sobrinho D., Lakshminarayanan B., Najeeb S., Nafna E.K., Marathakam A., Uddin M.S. (2020). Studies on the interaction between HSA and new halogenated metformin derivatives: Influence of lipophilic groups in the binding ability. J. Biomol. Struct. Dyn..

[20] Chaves O.A., Mathew B., Joy M., Lohidakshan K.K., Marathakam A., Netto-Ferreira J.C. (2018). Introduction of fluorinated environment on metformin. Evaluation of its serum-albumin interaction with molecular modeling studies. J. Mol. Liq..

[21] Tanwir A., Jahan R., Quadir M., Kaisar M.A., Hossain M.K. (2012). Spectroscopic Studies of the Interaction between Metformin Hydrochloride and Bovine Serum Albumin. Dhaka Univ. J. Pharm. Sci..

[22] Sharma D., Ojha H., Pathak M., Singh B., Sharma N., Singh A., Kakkar R., Sharma R.K. (2016). Spectroscopic and molecular modelling studies of binding mechanism of metformin with bovine serum albumin. J. Mol. Struct..

[23] Berbudi A., Rahmadika N., Tjahjadi A.I., Ruslami R. (2020). Type 2 Diabetes and its Impact on the Immune System. Curr. Diabetes Rev..

[24] Maity S., Leton N., Nayak N., Jha A., Anand N., Thompson K., Boothe D., Cromer A., Garcia Y., Al-Islam A. (2024). A systematic review of diabetic foot infections: Pathogenesis, diagnosis, and management strategies. Front. Clin. Diabetes Healthc..

[25] Javid A., Zlotnikov N., Pětrošová H., Tang T.T., Zhang Y., Bansal A.K., Ebady R., Parikh M., Ahmed M., Sun C. (2016). Hyperglycemia Impairs Neutrophil-Mediated Bacterial Clearance in Mice Infected with the Lyme Disease Pathogen. PLoS ONE.

[26] Pan X., Yu X., Qin P., Yu W. (2025). Molecular mechanism underlying the modulated toxicity of differently charged and sized nanoplastics by bovine serum albumin. J. Photochem. Photobiol. B Biol..

[27] Rajendran D., Chandrasekaran N. (2023). Unveiling the modification of esterase-like activity of serum albumin by nanoplastics and their cocontaminants. ACS Omega.

[28] Baysal A., Saygin H., Soyocak A. (2024). A Comparative Study on the Interaction Between Protein and PET Micro/Nanoplastics: Structural and Surface Characteristics of Particles and Impacts on Lung Carcinoma Cells (A549) and *Staphylococcus aureus*. Environ. Toxicol..

[29] Saygin H., Baysal A., Tilkili B., Apaydin E., Ozbek P. (2025). Understanding the PET micro/nanoplastics as an environmental stressor on pancreatin enzyme: Leaching and binding characterization by multi-spectroscopic and molecular docking examination, and the resulting impact on *Escherichia coli*. Nanotoxicology.

[30] Pańczyk T., Wolski P., Nieszporek K. (2025). Size-Dependent Interactions of Degraded PET Nanoparticles with Human Serum Albumin: Thermodynamic and Molecular Insights. J. Phys. Chem. B.

[31] Kihara S., Heijden NJvan der Seal C., Mata J., Whitten A.E., Köper I., McGillivray D.J. (2019). Soft and Hard Interactions between Polystyrene Nanoplastics and Human Serum Albumin Protein Corona. Bioconjugate Chem..

[32] Rajendran D., Chandrasekaran N. (2023). Journey of micronanoplastics with blood components. RSC Adv..

[33] Rajendran D., Varghese R.P., Doss C.G.P., Shivashankar M., Chandrasekaran N. (2023). Interaction of antidiabetic formulation with nanoplastics and its binary influence on plasma protein. Environ. Toxicol. Pharmacol..

[34] Pani B., Chandrasekaran N. (2023). Adsorption of clarithromycin on polystyrene nanoplastics surface and its combined adverse effect on serum albumin. Colloids Surf. B Biointerfaces.

[35] Ju P., Zhang Y., Zheng Y., Gao F., Jiang F., Li J., Sun C. (2020). Probing the toxic interactions between polyvinyl chloride microplastics and Human Serum Albumin by multispectroscopic techniques. Sci. Total Environ..

[36] Ju P., Zhang Y., Ding J., Zheng Y., Wang S., Jiang F., Sun C. (2021). New insights into the toxic interactions of polyvinyl chloride microplastics with bovine serum albumin. Environ. Sci. Pollut. Res..

[37] LaClair C.E., Etzel M.R. (2009). Turbidity and protein aggregation in whey protein beverages. J. Food Sci..

[38] Bianco V., Franzese G., Coluzza I. (2019). In Silico Evidence That Protein Unfolding is a Precursor of Protein Aggregation. ChemPhysChem.

[39] Casals E., Pfaller T., Duschl A., Oostingh G.J., Puntes V. (2010). Time Evolution of the Nanoparticle Protein Corona. ACS Nano.

[40] Mourdikoudis S., Pallares R.M., Thanh N.T.K. (2018). Characterization techniques for nanoparticles: Comparison and complementarity upon studying nanoparticle properties. Nanoscale.

[41] Cho E.J., Holback H., Liu C.K., Abouelmagd S.A., Park J., Yeo Y. (2013). Nanoparticle Characterization: State of the Art, Challenges, and Emerging Technologies. Mol. Pharm..

[42] Aminian M., Nabatchian F., Vaisi-Raygani A., Torabi M. (2012). Mechanism of Coomassie Brilliant Blue G-250 binding to cetyltrimethylammonium bromide: An interference with the Bradford assay. Anal. Biochem..

[43] Walker J.M. (2009). The Protein Protocols Handbook.

[44] Charrier J.G., Anastasio C. (2012). On dithiothreitol (DTT) as a measure of oxidative potential for ambient particles: Evidence for the importance of soluble transition metals. Atmos. Chem. Phys..

[45] Jiang H., Ahmed C.M.S., Canchola A., Chen J.Y., Lin Y.H. (2019). Use of dithiothreitol assay to evaluate the oxidative potential of atmospheric aerosols. Atmosphere.

[46] Figueroa D., Asaduzzaman M., Young F. (2018). Real time monitoring and quantification of reactive oxygen species in breast cancer cell line MCF-7 by 2′,7′-dichlorofluorescin diacetate assay. J. Pharmacol. Toxicol. Methods.

[47] Tabata F., Wada Y., Kawakami S., Miyaji K. (2021). Serum albumin redox states: More than oxidative stress biomarker. Antioxidants.

[48] Masadeh M.M., Alzoubi K.H., Al-Azzam S.I., Khabour O.F., Al-Batayneh K.M. (2021). Metformin as a potential adjuvant antimicrobial agent against multidrug resistant bacteria. Clin. Pharmacol. Adv. Appl..

[49] Temel A., Ateş A., Aksoyalp Z.Ş. (2026). Effects of empagliflozin and metformin on biofilm formation and pathogenicity factors of urinary *Escherichia coli* isolates. Folia Microbiol..

[50] Xu T., Cao W., Fan S., Liu R., Zhu H., Lu X., Zhang Z., He X., Zhang K., Huang J. (2026). Repurposing metformin as a dual-function agent to combat *E. coli*-induced mastitis: Mechanistic insights into biofilm dispersion and AMPK/SIRT1-mediated NF-κB inhibition. PLoS Pathog..

[51] Abbas H.A., Shaker G.H., Mosallam F.M., Gomaa S.E. (2022). Novel silver metformin nano-structure to impede virulence of *Staphylococcus aureus*. AMB Express.

[52] Imlay J.A. (2013). The molecular mechanisms and physiological consequences of oxidative stress: Lessons from a model bacterium. Nat. Rev. Microbiol..

[53] Seixas A.F., Quendera A.P., Sousa J.P., Silva A.F.Q., Arraiano C.M., Andrade J.M. (2022). Bacterial response to oxidative stress and RNA oxidation. Front. Genet..

[54] Posada A.C., Kolar S.L., Dusi R.G., Francois P., Roberts A.A., Hamilton C.J., Liu G.Y., Cheung A. (2014). Importance of bacillithiol in the oxidative stress response of *Staphylococcus aureus*. Infect. Immun..

[55] Perera V.R., Newton G.L., Pogliano K. (2015). Bacillithiol: A key protective thiol in *Staphylococcus aureus*. Expert Rev. Anti-Infect. Ther..

[56] Jia J., Liu Q., Zhao E., Li X., Xiong X., Wu C. (2024). Biofilm formation on microplastics and interactions with antibiotics, antibiotic resistance genes, and pathogens in aquatic environments. Eco-Environ. Health.

[57] Kadac-Czapska K., Ośko J., Knez E., Grembecka M. (2024). Microplastics and oxidative stress—Current problems and prospects. Antioxidants.

[58] Chen H., Lv X., Zhao C., Zheng L., Zhang H., Hao Y., Li P., Wang X., Zheng Z. (2025). Research progress in mechanisms of neurotoxicity induced by micro(nano)plastic exposure. All Life.

[59] da Cruz Nizer W.S., Adams M.E., Allison K.N., Montgomery M.C., Mosher H., Cassol E., Overhage J. (2024). Oxidative stress responses in biofilms. Biofilm.

[60] Gaupp R., Ledala N., Somerville G.A. (2012). Staphylococcal response to oxidative stress. Front. Cell. Infect. Microbiol..

[61] Saygin H., Baysal A. (2026). Micro/Nanoplastic-Enhanced Oxidative Potential, Antioxidant Depletion, Inflammation in PM_2.5_ and Cytologic and Metabolomic Shifts. Microplastics.

[62] Arora S., Jain J., Rajwade J.M., Paknikar K.M. (2008). Cellular responses induced by silver nanoparticles: In vitro studies. Toxicol. Lett..

[63] Saygin H., Baysal A. (2022). Single and combined effects of antibiotics and nanoplastics from surgical masks and plastic bottles on pathogens. Comp. Biochem. Physiol. Part C Toxicol. Pharmacol..

