# Supplementary material for: PET Micro(nano)plastics Modulate Metformin–Albumin Binding and Species-Specific Bacterial Responses
Source: Int J Mol Sci. 2026 Aug 21;27(16):7504. doi: 10.3390/ijms27167504 (PMC13513656; doi:10.3390/ijms27167504)
Supplement: Supplementary file 1 [file ijms-27-07504-s001.zip › ijms-4460916-supplementary.pdf]

Supplementary Materials

Supplementary Figure S1. Metformin-only baseline responses of E. coli and S. aureus across the investigated bacterial endpoints. The left column shows E. coli and the right column shows S. aureus responses for OD600, ROS, SOD, GSH, lipid peroxidation (LPO), and biofilm formation at Met1×, Met5×, Met10×, Met20×, and Met50×. Values were normalized to the corresponding untreated bacterial control, which was set to 1.0.

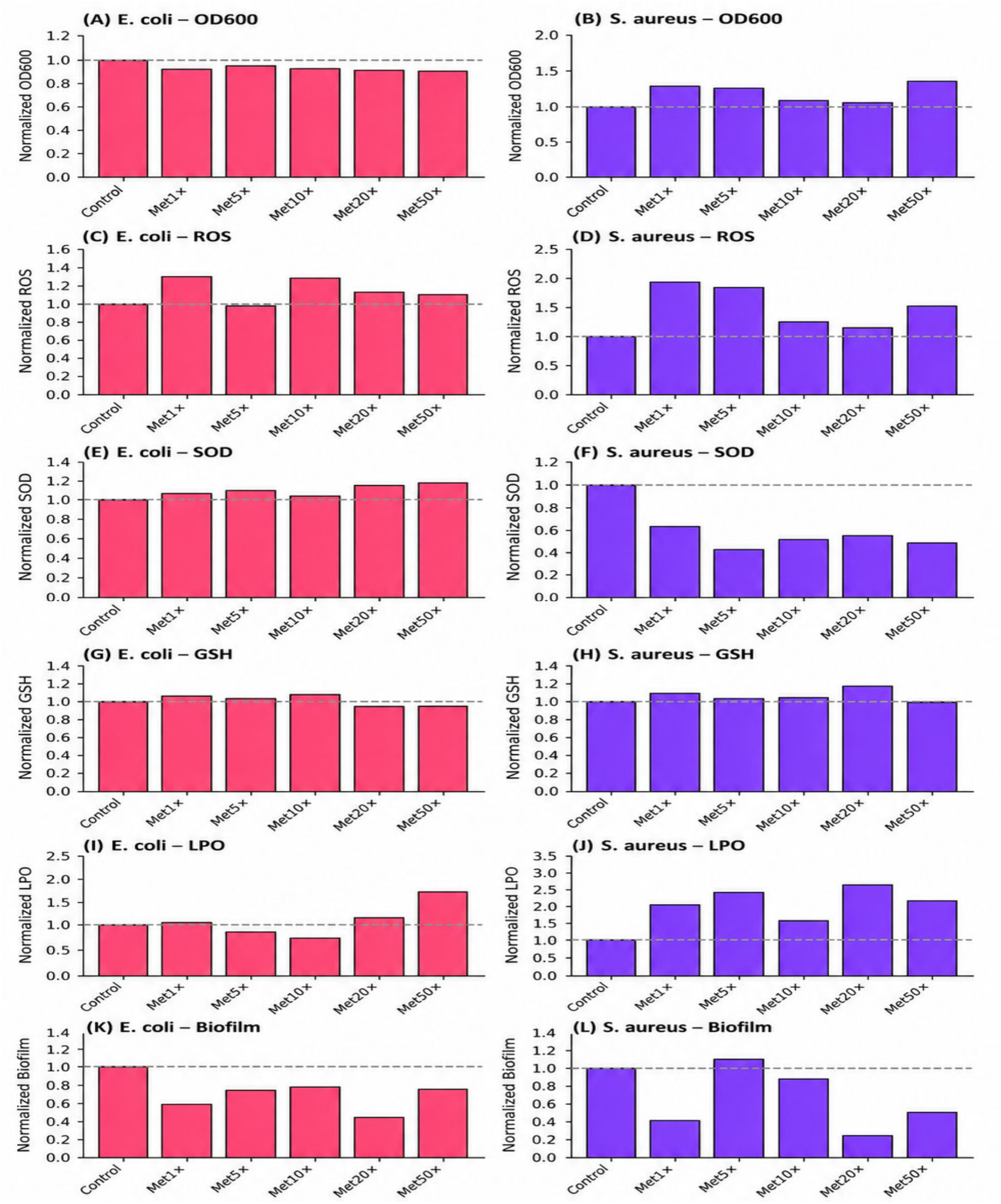

Supplementary Figure S2. Physicochemical parameters of tested PET MNPs [11,28]

(a) (b) (c)

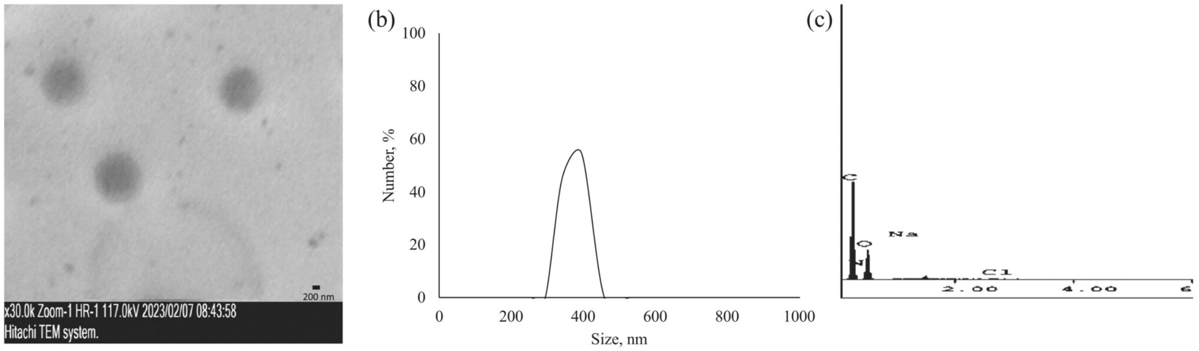

(d) (e)

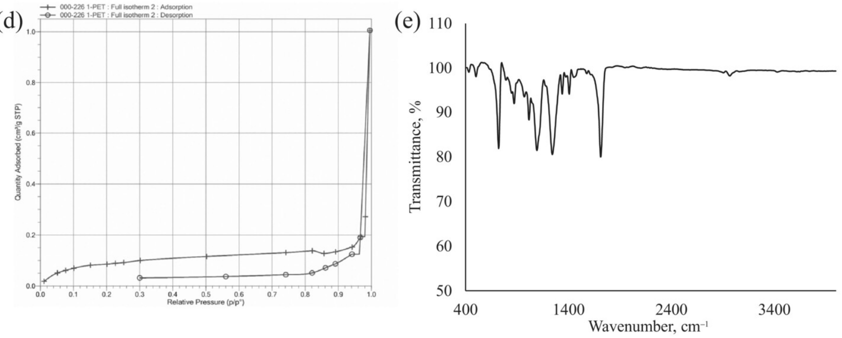

Parameter	Value
Zeta potentials, mV	−11.3
BET Surface Area, m²/g	0.3257
Micropore Area, m²/g	0.0027
